# Identification of quantitative trait loci (QTL) and meta-QTL analysis for kernel size-related traits in wheat (*Triticum aestivum* L.)

**DOI:** 10.1186/s12870-022-03989-9

**Published:** 2022-12-23

**Authors:** Jingfu Ma, Yuan Liu, Peipei Zhang, Tao Chen, Tian Tian, Peng Wang, Zhuo Che, Fahimeh Shahinnia, Delong Yang

**Affiliations:** 1 State Key Lab of Aridland Crop Science, Lanzhou, Gansu, China; 2 College of Agronomy, Gansu Agricultural University, Lanzhou, Gansu, China; 3 College of Life Science and Technology, Gansu Agricultural University, Lanzhou, Gansu, China; 4 Plant Seed Master Station of Gansu Province, Lanzhou, Gansu, China; 5 Institute for Crop Science and Plant Breeding, Bavarian State Research Centre for Agriculture, Freising, Germany

**Keywords:** Wheat, Kernel size, MQTL analysis, Gene expression, Candidate gene

## Abstract

**Background:**

Kernel size-related traits, including kernel length (KL), kernel width (KW), kernel diameter ratio (KDR) and kernel thickness (KT), are critical determinants for wheat kernel weight and yield and highly governed by a type of quantitative genetic basis. Genome-wide identification of major and stable quantitative trait loci (QTLs) and functional genes are urgently required for genetic improvement in wheat kernel yield. A hexaploid wheat population consisting of 120 recombinant inbred lines was developed to identify QTLs for kernel size-related traits under different water environments. The meta-analysis and transcriptome evaluation were further integrated to identify major genomic regions and putative candidate genes.

**Results:**

The analysis of variance (ANOVA) revealed more significant genotypic effects for kernel size-related traits, indicating the moderate to high heritability of 0.61–0.89. Thirty-two QTLs for kernel size-related traits were identified, explaining 3.06%—14.2% of the phenotypic variation. Eleven stable QTLs were detected in more than three water environments. The 1103 original QTLs from the 34 previous studies and the present study were employed for the MQTL analysis and refined into 58 MQTLs. The average confidence interval of the MQTLs was 3.26-fold less than that of the original QTLs. The 1864 putative candidate genes were mined within the regions of 12 core MQTLs, where 70 candidate genes were highly expressed in spikes and kernels by comprehensive analysis of wheat transcriptome data. They were involved in various metabolic pathways, such as carbon fixation in photosynthetic organisms, carbon metabolism, mRNA surveillance pathway, RNA transport and biosynthesis of secondary metabolites.

**Conclusions:**

Major genomic regions and putative candidate genes for kernel size-related traits in wheat have been revealed by an integrative strategy with QTL linkage mapping, meta-analysis and transcriptomic assessment. The findings provide a novel insight into understanding the genetic determinants of kernel size-related traits and will be useful for the marker-assisted selection of high yield in wheat breeding.

**Supplementary Information:**

The online version contains supplementary material available at 10.1186/s12870-022-03989-9.

## Background

Wheat (*Triticum aestivum* L.) is one of the most important cereal crops worldwide, providing nearly 20% of the calories for the world population [1]. It is estimated that wheat yield needs to be increased by 70% to meet the food demand associated with the growth of the world population [2]. In this context, improving wheat yield is critical to ensuring food security in the future. Wheat yield is significantly influenced by thousand kernel weight (TKW), kernel number per spike (KNS), and spike number per unit area (SN) [3, 4]. Of these, TKW has been selected as an essential trait in wheat breeding programs, due to its high heritability [5]. Kernel size-related traits, as one of the critical factors determining the formation of kernel weight, are mainly composed of kernel length (KL), kernel width (KW), kernel diameter ratio (KDR) and kernel thickness (KT) [6]. Larger kernels positively influence wheat seedling growth and significantly contribute to high-yield improvement [4, 7, 8]. Therefore, deciphering the genetic basis and finding functional genes for kernel size are critical for the enhancement of grain yield traits in wheat.

Grain-size related traits have attracted considerable attention in wheat breeding. Yield-related traits are complex quantitative traits controlled by polygenes [9–11], which are strongly influenced by genotypic and environmental factors [12]. In the last two decades, a large number of QTLs underlying wheat kernel size-related traits have been successfully identified by traditional bi-parental linkage mapping [7, 9–11, 13–17] and genome-wide association studies (GWAS) [18–23]. However, due to the large and highly repetitive nature of the wheat genome, identifying stable and robust QTLs for kernel size-related and yield traits remains challenging in wheat breeding [24, 25].

Previous studies reported that QTLs for grain size were generally mapped in large confidence interval (CI) with minor effects and are significantly influenced by different genetic backgrounds and environments, which limits the usefulness of these QTLs in wheat breeding programs [26]. The meta-QTL analysis is a robust method for the genetic analysis of complex traits by integrating QTLs from different studies to obtain stable genetic regions controlling a quantitative trait [27]. Compared to QTLs identified in a single study, MQTLs have the advantage of a smaller CI and a higher consistency under different genetic backgrounds. The meta-QTL analysis also facilitates the identification of candidate genes in a genome as complex as wheat.

MQTL analysis has been successfully applied in various crops, including maize [28–31], rice [26, 32, 33] and soybean [34]. MQTL analysis in wheat has also been effectively used to establish the consensus map of QTLs for many agronomic traits [35–37]. Previous studies integrated QTLs for yield and yield-related traits from published articles. They identified 12 significant MQTLs on chromosomes 1A, 1B, 2A, 2D, 3B, 4A, 4B, 4D and 5A, including two critical underlying genes, *Rht* and *Vrn* [38]. Tyagi et al. (2015) performed a meta-analysis of QTLs associated with kernel morphological traits and mapped 17 MQTLs on seven chromosomes in wheat [39]. In a previous study, a total of 2230 QTLs for yield and yield-related traits were used for meta-QTL analysis and 145 MQTLs were identified, of which 85 were verified by GWAS using different natural populations. Within 76 MQTL core intervals, 237 candidate genes involved in photoperiod response, kernel development, multiple plant growth regulatory pathways, carbon and nitrogen metabolism and ear and flower organ development were identified through searching for sequence homology and expression analysis [37]. Meanwhile, Liu et al. (2020) performed a meta-analysis with 381 QTL related to yield and identified 86 MQTL and 210 candidate genes in wheat [40]. In addition to yield-related traits, MQTL analysis was also used to discover consistent QTLs and identify candidate genes for various quantitative traits such as leaf rust [41], drought and heat tolerance [42–44], salt tolerance [45] and disease resistance [46–48].

The present study used the inclusive composite interval mapping (ICIM) method to identify the QTLs controlling kernel size-related traits across seven environments. We performed a meta-analysis by combining the QTLs detected in our study with the 1071 QTLs from previous studies. Our main objectives were to: (1) identify stable QTL for traits related to kernel size in seven environments; (2) discover and map MQTLs from numerous reported QTL and current studies; and (3) identify candidate genes related to kernel size associated with MQTL intervals.

## Results

### Phenotypic and correlation analyses

**Fig. 1:**
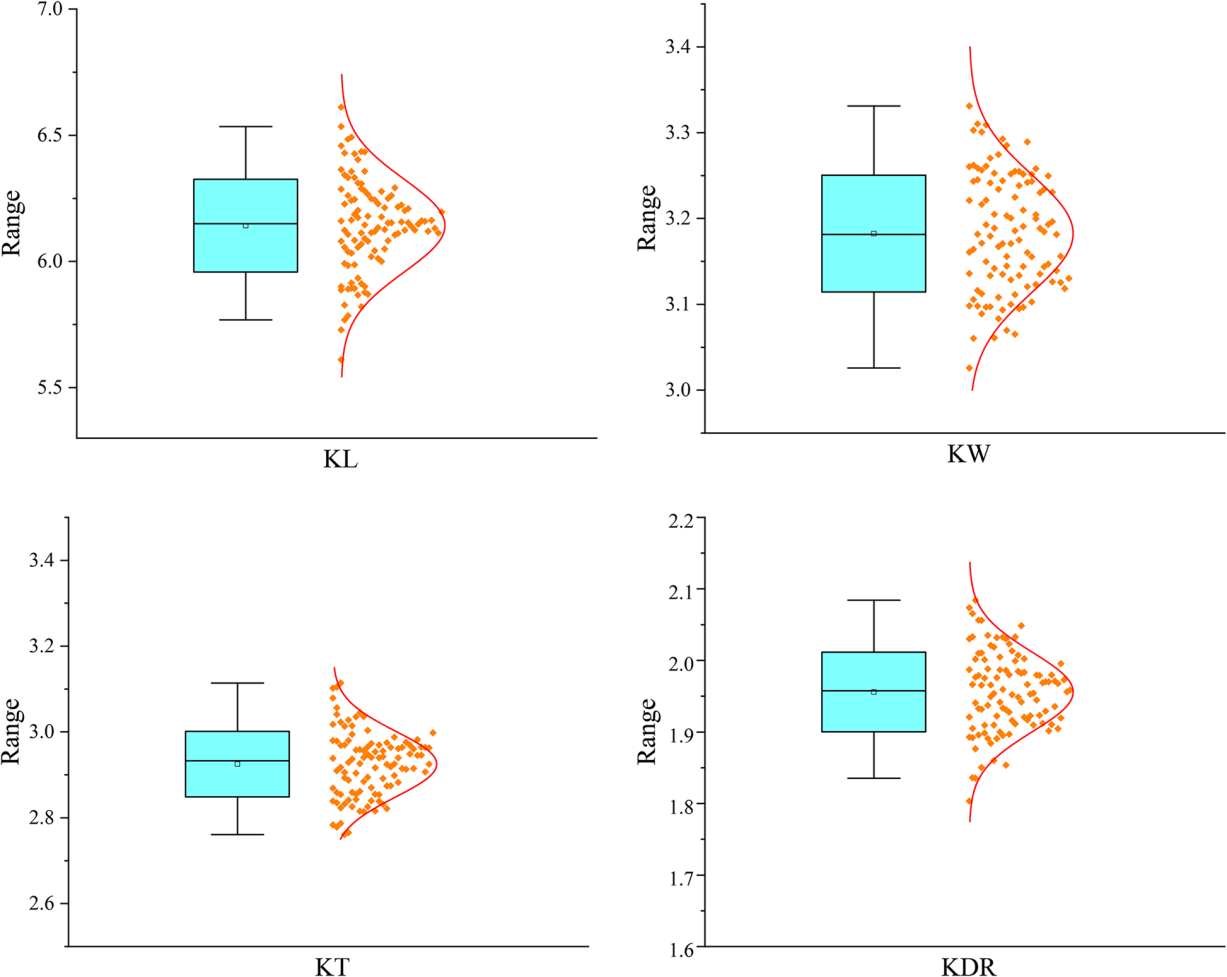
The frequency distribution of kernel size-related traits

In the field trials conducted in seven environments (E1-E7), the parental line Q9086 had a significantly longer and wider kernel than Longjian19 (Table S1). In KT, the parental line Longjian19 had an advantage over Q9086. In the RILs population, all traits varied widely and had an approximately normal distribution with significantly transgressive segregation (Fig. 1). The coefficients of variation for KL, KW, KDR and KT ranged from 3.47% to 5.71%, 2.47% to 6.27%, 3.24% to 8.57% and 3.88% to 5.45%, respectively. The ANOVA of four kernel size-related traits revealed significant differences (*P* < 0.01) in the variation factors of environment, genotype, and genotype × environment interaction. Among the kernel size-related traits, KL (*h*^*2*^ = 0.89) and KDR (*h*^*2*^ = 0.70) were highly heritable, followed by KW (*h*^*2*^ = 0.67) and KT (*h*^*2*^ = 0.61) (Table S2).

**Fig. 2:**
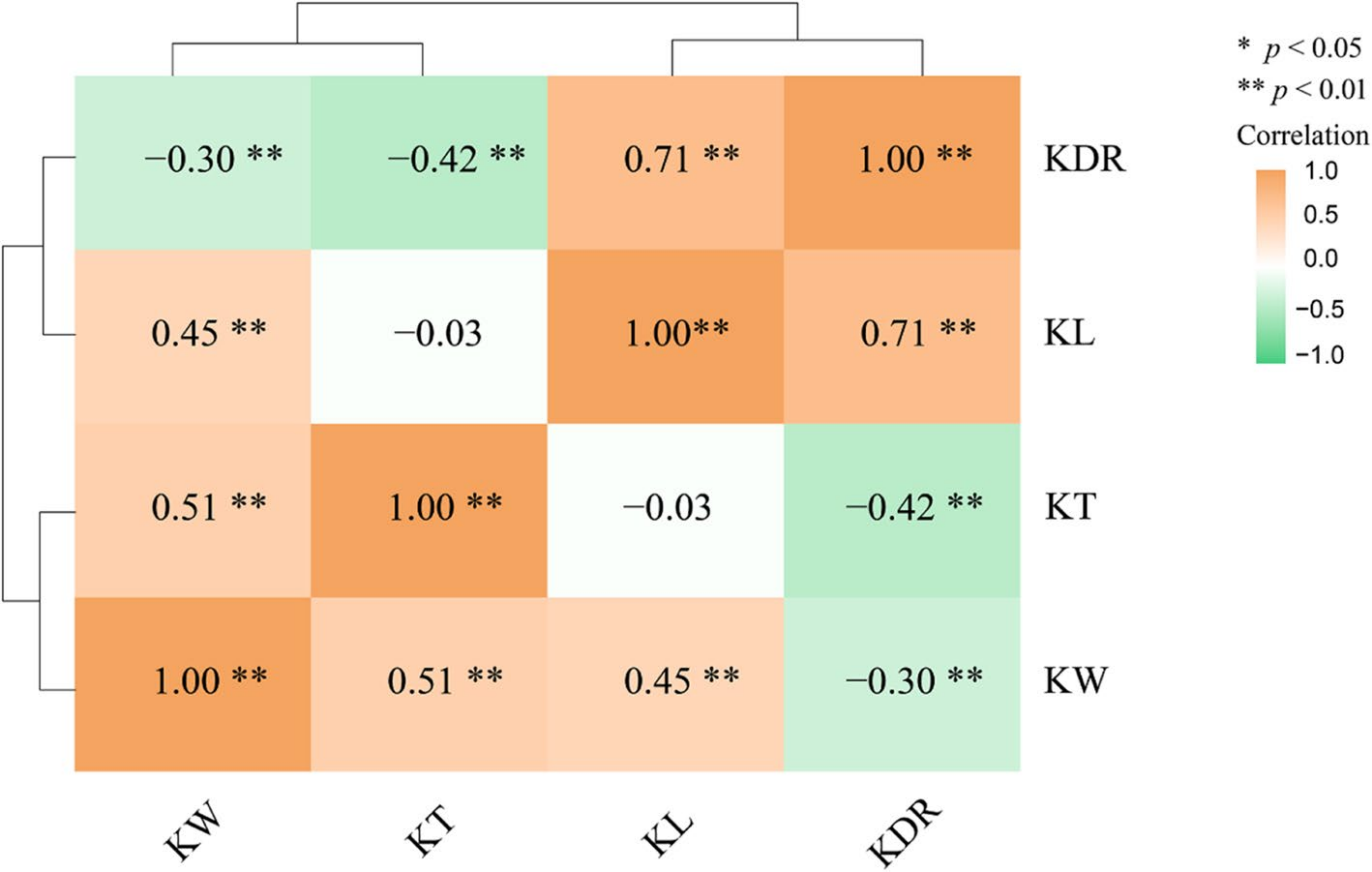
Correlation coefficient among four kernel size-related traits in the Q9086/Longjian19 RILs population. * and ** indicate significant level at *P* < 0.05 and *P* < 0.01, respectively

Significant correlations were found among KL, KW, KDR and KT (Fig. 2). KL showed a positive correlation with KW (*r* = 0.45, *P* < 0.01) and KDR (*r* = 0.71, *P* < 0.01), whereas there was a negative correlation with KT (*r* = -0.03, *P* < 0.05). KW showed a significant positive correlation with KT (*r* = 0.41, *P* < 0.01) and a negative correlation with KDR (*r* = -0.30, *P* < 0.05). In addition, a negative correlation was observed between KT and KDR (*r* = -0.42, *P* < 0.01).

### QTLs controlling kernel size-related traits

**Fig. 3:**
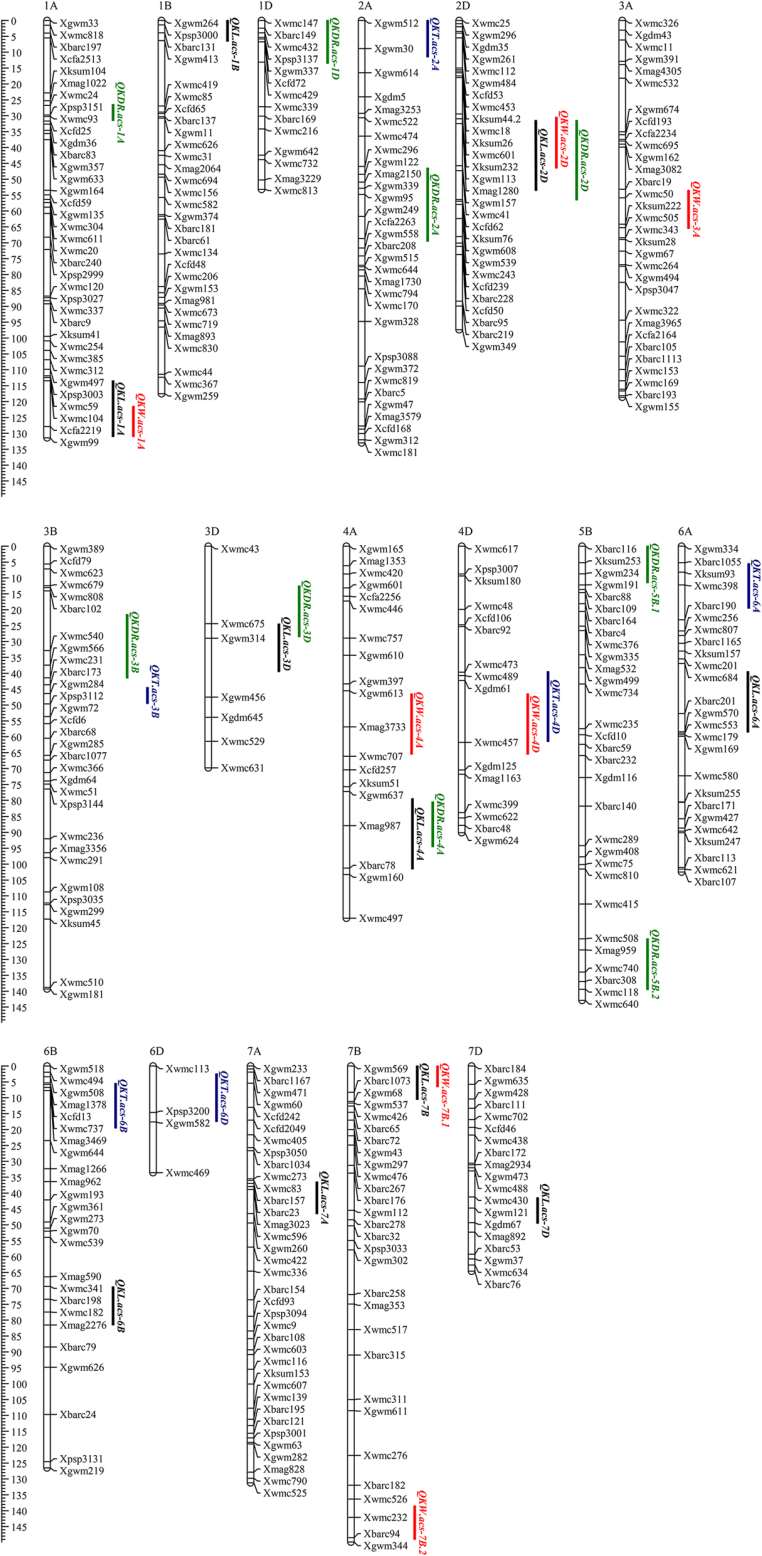
Chromosomal locations of QTLs detected for kernel size-related traits. The vertical bars with different colors represent the interval of QTLs for kernel length (black), kernel width (red), kernel diameter ratio (green), and kernel thickness (blue)

QTL mapping detected 32 QTLs for kernel size-related traits with the PVE ranging from 3.06% to 14.2% in different environments (Table S3, Fig. 3). These loci were mapped on 17 chromosomes, except for chromosomes 2B, 4B, 5A and 5D. Eleven stable QTLs, namely *QKL.acs-1A*, *QKW.acs-1A*, *QKDR.acs-2A*, *QKL.acs-2D*, *QKW.acs-3A*, *QKDR.acs-4A*, *QKDR.acs-5B.2*, *QKL.acs-6A*, *QKL.acs-6B*, *QKW.acs-7B.1* and *QKW.acs-7B.2*, were detected in more than three environments, with PVE ranging from 3.07% to 9.85%.

Ten QTLs associated with KL were identified on chromosomes 1A, 1B, 2D, 3D, 4A, 6A, 6B, 7A, 7B and 7D, with PVE ranging from 3.40% to 8.26% (Table S3, Fig. 3). Of these, four stable QTLs were identified for KL on chromosomes 1A, 2D, 6A and 6B, including *QKL.acs-1A* identified in E3, E4 and E5, *QKL.acs-2D* identified in E3, E4 and E7, *QKL.acs-6A* identified in E3, E5 and E7, *QKL.acs-6B* identified in E1, E2, E3, E6 and E7, respectively. Notably, *QKL.acs-6B*, with 4.07%-8.26% of the PVE, was detected in five environments (E1, E2, E3, E6 and E7). Except for the QTL *QKL.acs-6B*, the additive effect of the other three stable QTLs contributed to decreasing KL.

Among the seven QTLs associated with KW on six chromosomes (1A, 2D, 3A, 4A, 4D and 7B), with PVEs ranged from 3.35% to 9.85% (Table S3, Fig. 3). Four stable QTLs, *QKW.acs-1A* identified in E4, E6 and E7, *QKW.acs-3A* identified in E2, E3 and E6, *QKW.acs-7B.1* identified in E4, E5 and E6, and *QKW.acs-7B.2* identified in E1, E5 and E6, which were mapped on chromosomes 1A, 3A and 7B, respectively. *QKW.acs-1A* and *QKW.acs-7B.1* had a negative additive effect on KW, while *QKW.acs-3A* and *QKW.acs-7B.2* showed a positive additive effect for increasing KW. The QTLs *QKW.acs-7B.1* and *QKW.acs-7B.2* were detected on the same chromosomes with opposite additive effects.

Nine QTLs for KDR were mapped on chromosomes 1A, 1D, 2A, 2D, 3B, 3D, 4A and 5B, with individual PVE ranging from 3.06% to 14.2% (Table S3, Fig. 3). Three stable QTLs, *QKDR.acs-2A* identified in E1, E5 and E7, *QKDR.acs-4A* identified in E2, E3, E6 and E7, and *QKDR.acs-5B.2* identified in E1, E3 and E5, were also detected in at least three environments with a range of PVE from 3.06% to 6.9%. A major QTL (*QKDR.acs-2D*) was identified and explained 14.2% of phenotypic variance. In addition, a stable QTL *QKDR.acs-4A* was detected in four environments (E2, E3, E6 and E7) and accounted for 3.06–6.9% of the PVE.

On chromosomes 2A, 3B, 4D, 6A, 6B and 6D, six QTLs associated with KT were identified, each accounting for 4.6%-10% of PVE (Table S3, Fig. 3). They were all detected in less than two environments. Of those, QTL *QKT.acs-3B.1* owned the highest PVE of 10%.

### QTLs identified under different water environments

In the present study, 23 QTLs for kernel size-related traits were detected under DS and WW environments (Table S3, Fig. 3). Under DS conditions, 14 QTLs were located on chromosomes 1A, 1B, 1D, 2D, 3B, 3D, 4A, 5B, 6A, 6B and 7D with PVE ranging from 3.4% to 14.2%. Two stable QTLs, *QKL.acs-2D* and *QKW.acs-1A*, were identified under DS conditions. Under WW environments, nine QTLs for kernel size-related traits were located on chromosomes 1A, 2A, 3D, 4A, 4D, 6D, 7A and 7B with PVE ranging from 4.23% to 9.53%. Importantly, nine stable QTLs, including *QKL.acs-1A*, *QKDR.acs-2A*, *QKW.acs-3A*, *QKDR.acs-4A*, *QKDR.acs-5B.2*, *QKL.acs-6A*, *QKL.acs-6B*, *QKW.acs-7B.1* and *QKW.acs-7B.2*, were identified under both WW and DS environments.

### Initial QTLs collection for wheat kernel size-related traits

**Fig. 4:**
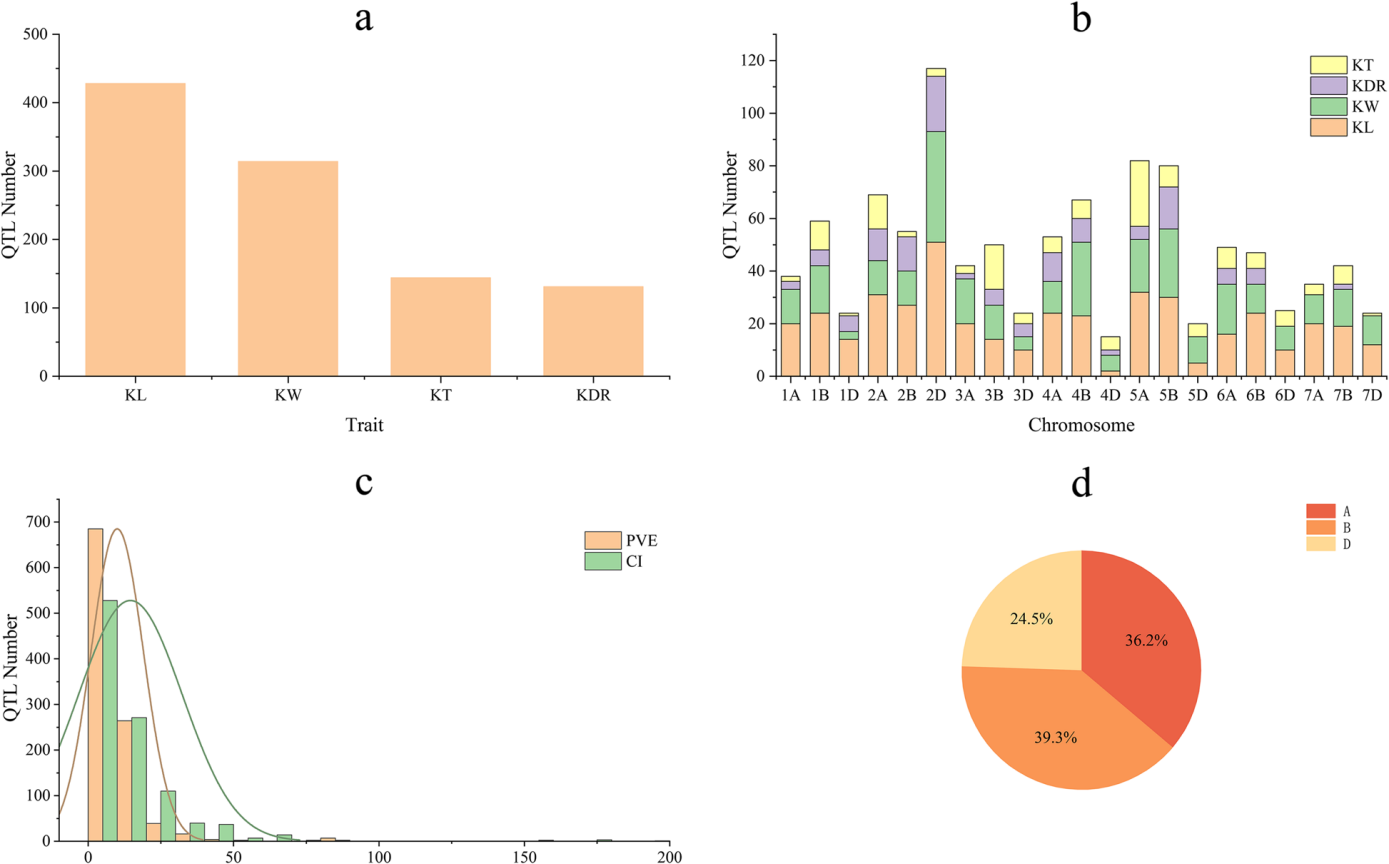
Number of QTLs collected (a) by trait category, KL (kernel length), KW (kernel width), KT (kernel thickness), and KDR (kernel diameter ratio) and (b) in 21 wheat chromosomes (c) frequencies of QTLs with different PVE (%) and CI values and (d) proportion of QTL numbers in wheat sub-genomes A, B, and D

By integrating 1071 initial QTLs from 34 QTL studies published between 2007 and 2020 (Table S4) and 32 QTLs identified in this study, a total of 1103 initial QTLs for kernel size-related traits were used for MQTL analysis (Fig. 4a). The distribution of initial QTLs significantly differed from homoeologous groups, sub-genomes and individual chromosomes. For example, the number of identified QTLs ranged from 101 on homoeologous group VII to 241 on group II, and from 15 on chromosome 4D to 117 on chromosome 2D (Fig. 4b). Of the 1103 initial QTLs, 399, 433 and 271 QTLs were distributed among sub-genomes A, B and D, respectively (Fig. 4d). The CI ranged from 0.14 cM to 190 cM, with an average of 14.52 cM (Fig. 4c). The proportion of phenotypic variance explained by individual QTL ranged from 1.00% to 86.31%, with an average of 9.98% (Fig. 4c).

### MQTL analysis for wheat kernel size-related traits

**Fig. 5:**
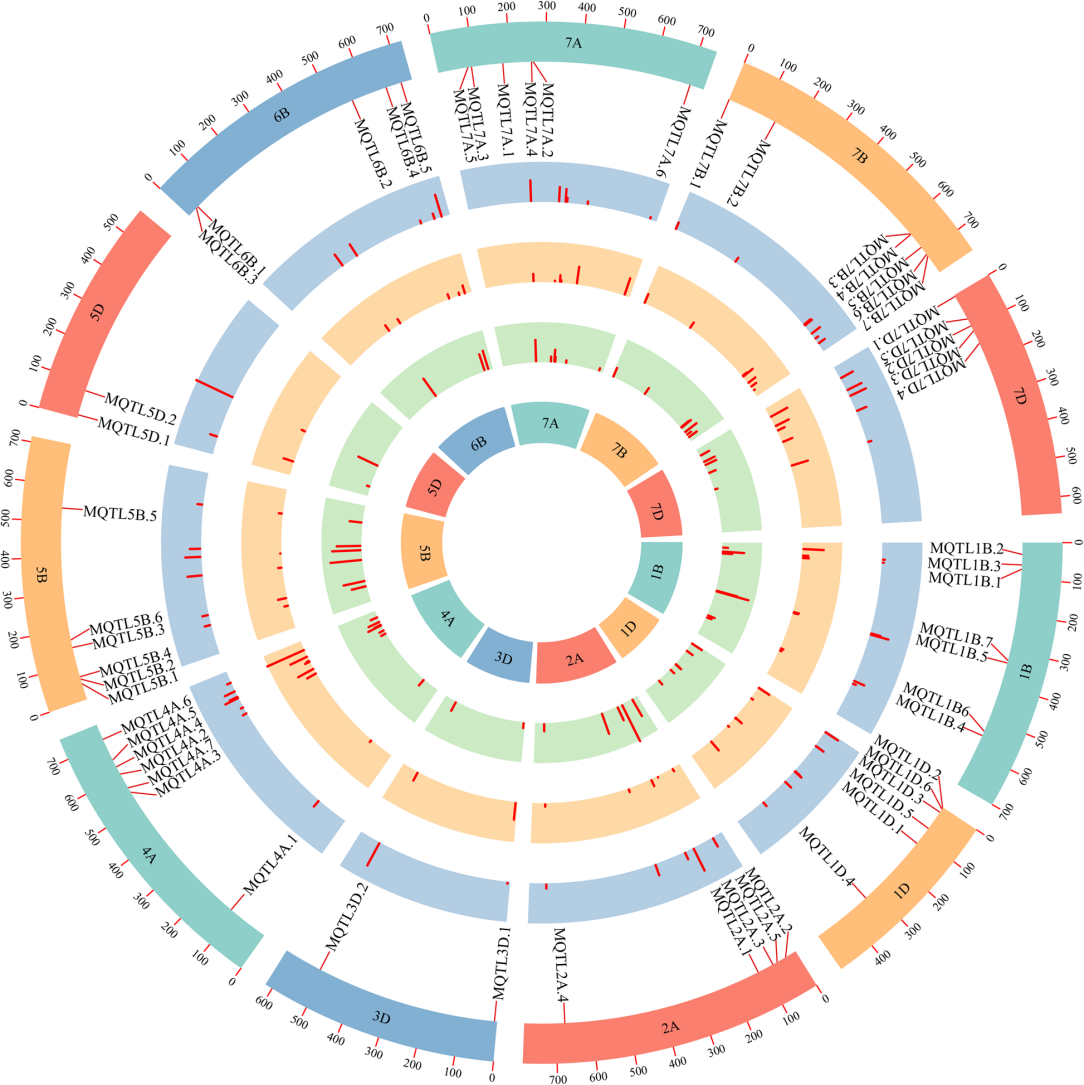
The chromosome distribution of the 58 MQTL for kernel size-related traits on 11 chromosomes. The circles from inside to outside represent the high-density consensus genetic map, the number of initial QTLs mapped on the MQTL interval, values of the confidence interval, values of the phenotypic variation explained, and the physical map, respectively

**Fig. 6:**
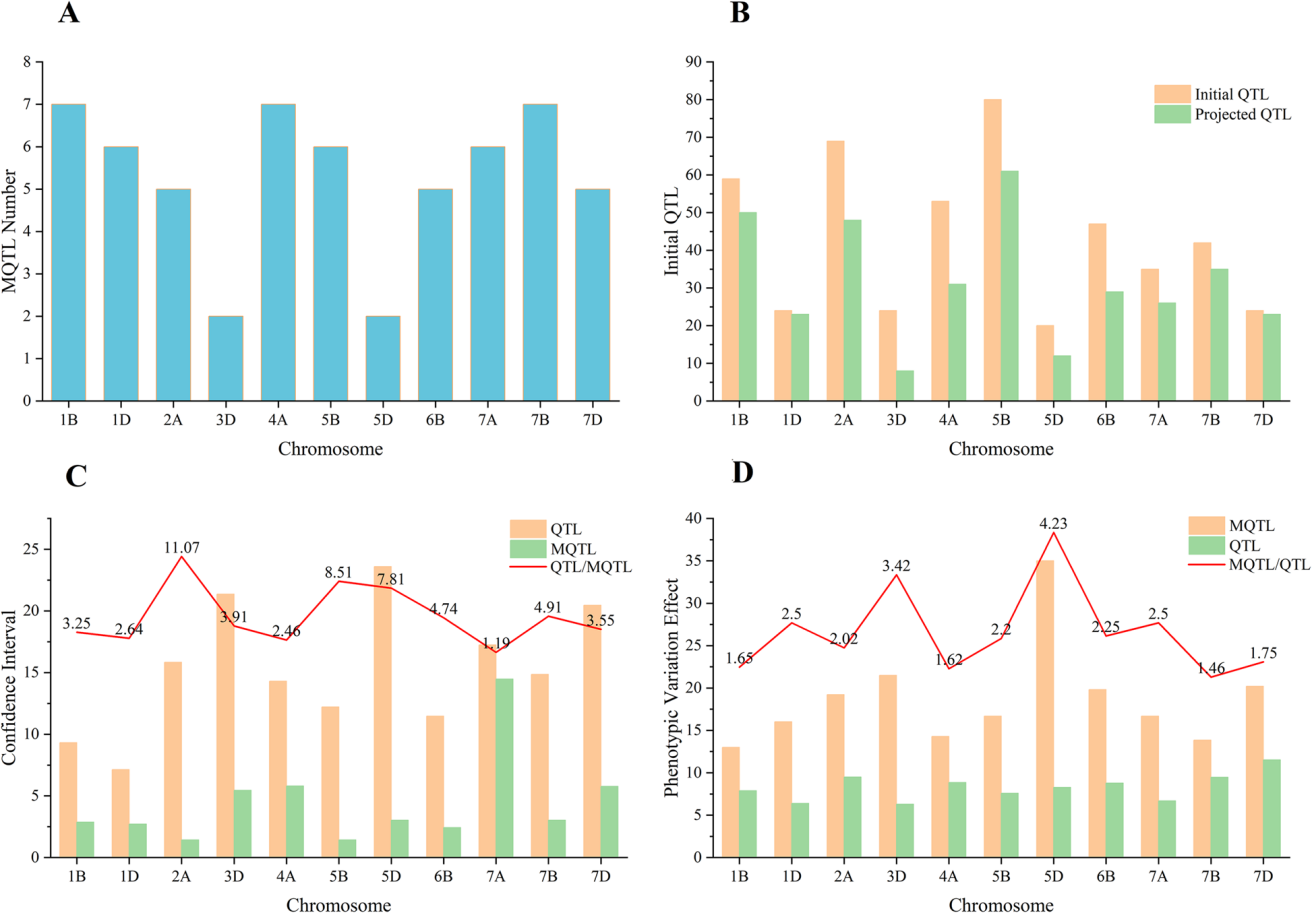
Number of MQTLs on different wheat chromosomes **(a)**; comparison of the original QTLs and the projected QTLs located in the MQTL intervals on different chromosomes **(b)**; comparison between the mean CI of the original QTLs and the MQTLs **(c)**; comparison between the mean PVE of the MQTLs and the original QTLs **(d)**. The numbers above the bars show the rate of change for the mean CI and PVE between the MQTLs and the original QTLs in **(c)** and **(d)**, respectively

A total of 346 initial QTLs were projected on the consensus map, while the remaining QTLs were eliminated due to the lack of common markers with the consensus map (Fig. 5). After meta-analysis, 58 MQTLs were detected on chromosomes 1B, 1D, 2A, 3D, 4A, 5B, 5D, 6B, 7A, 7B and 7D (Table S5). Each chromosome harbored two (3D) to seven MQTLs (1B, 4A and 7B) (Fig. 6a). The projected initial QTLs on the chromosomes varied from 20 (5D) to 80 (5B) (Fig. 6b). Most of the MQTL regions were co-localized for more than two kernel size-related traits (Fig. 5). The number of individual QTL per MQTL ranged from 1 (*MQTL6B.2* and *MQTL6B.3*) to 18 (*MQTL2A.5*) (Table S5). MQTL intervals ranged from 0.21 cM (*MQTL5B.6*) to 72.64 cM (*MQTL7A.6*) with an average of 4.46 cM, indicating a reduction in CI of 3.26 fold compared to the initial QTLs (14.54 cM) (Table S5, Fig. 6c). The PVE ranged from 5% (*MQTL1B.7*) to 56% (*MQTL5D.2*) with an average PVE of 17.12%, which was increased 1.72 fold (Table S5, Fig. 6d). Based on the comparison of the flanking marker sequences, the MQTLs had unique physical positions in the reference sequence of the Chinese Spring wheat genome. The physical interval of these 58 MQTLs ranged from 1.54 Kb to 580.66 Mb (Table S5). Of these, 12 MQTLs with a physical interval less than 20 Mb were selected as core MQTLs.

### Candidate genes mining and expression analysis

We identified 1864 potential candidate genes in 12 core MQTL intervals, with the lowest (1) and highest (487) number of potential candidate genes in the *MQTL7B.4* and *MQTL2A.2* intervals, respectively. The potential candidate genes within the regions of 12 MQTLs were screened and annotated based on IWGSC RefSeq v1.1 from the Chinese Spring wheat reference genome (Table S6).

**Fig. 7:**
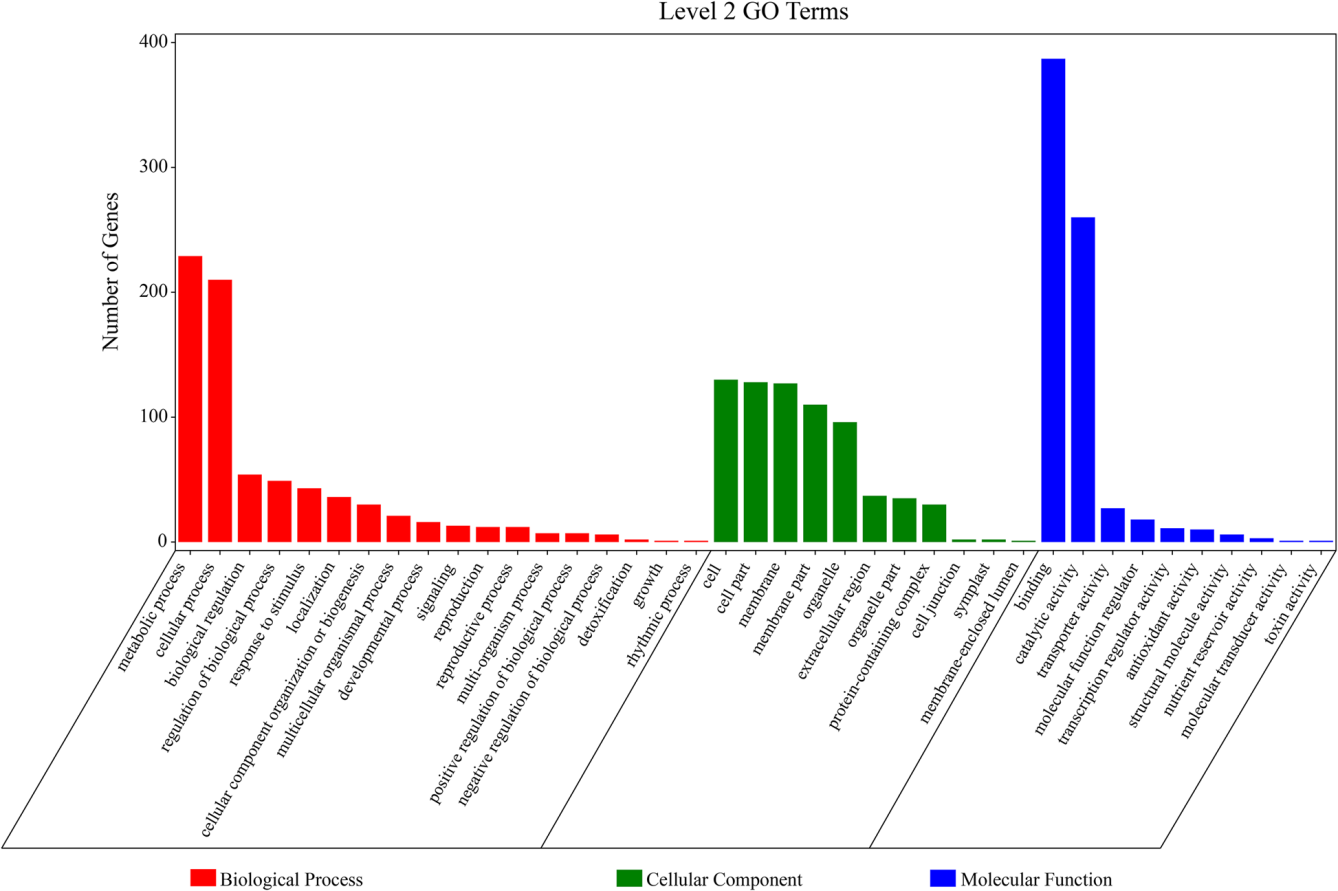
Gene ontology (GO) terms for 1863 candidate genes underlying MQTLs interval for kernel size-related traits

**Fig. 8:**
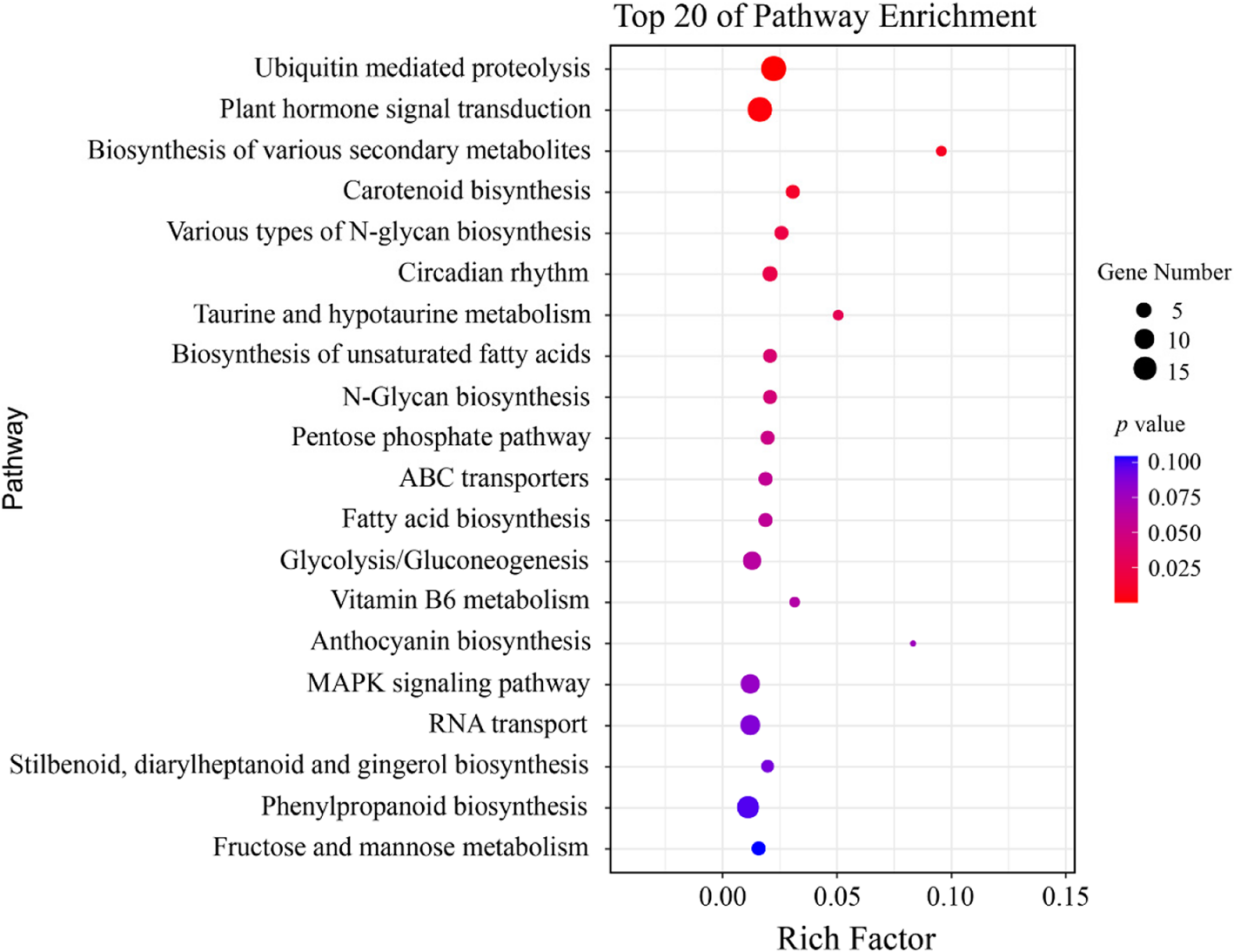
KEGG pathway enrichment of 1863 candidate genes

The GO terms associated with biological processes belonged to metabolic and cellular (229 and 210 potential candidate genes, respectively) pathways (Fig. 7). GO terms associated with molecular function were related to binding and catalytic activity (380 and 260 potential candidate genes, respectively). Regarding the cellular component, potential candidate genes were mainly related to the cell and cell part, with 130 and 128 potential candidate genes, respectively. KEGG analysis for potential candidate genes revealed that ubiquitin-mediated proteolysis and plant hormone signaling are the two most important pathways involved in the metabolic process (Fig. 8).

**Fig. 9:**
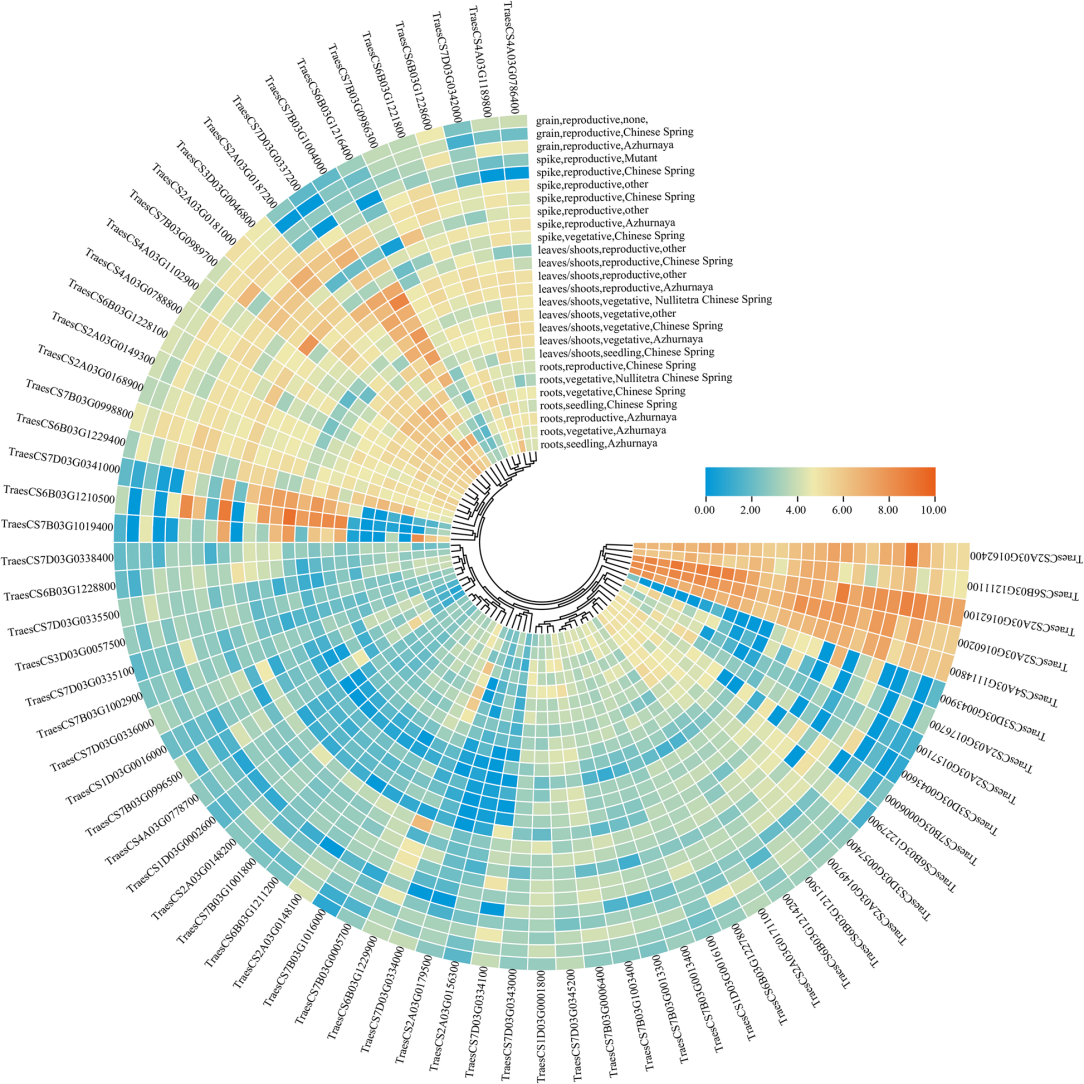
Heatmap showing the differential expression level of 70 candidate genes underlying MQTL intervals

The potential candidate genes were subjected to in silico expression analysis using RNAseq data [35–37]. Only 70 candidate genes predicted within the regions of nine MQTLs (except *MQTL7B.4*, *MQTL7B.5* and *MQTL7D.2*) were differentially expressed in spike and grain (Table 1, Fig. 9). These candidate genes are involved in various metabolic pathways, such as carbon fixation in photosynthetic organisms (4 genes), carbon metabolism (6 genes), mRNA surveillance pathway (4 genes), RNA transport (4 genes) and biosynthesis of secondary metabolites (18 genes).

**Table 1:** Identification of 70 candidate genes located in the nine core MQTL intervals

MQTL	Gene ID	Gene Position	Description	Orthology
MQTL1D.1	TraesCS1D02G004900	2,218,794–2,230,403	Paired amphipathic helix protein Sin3	NA
	TraesCS1D02G005200	2,468,742–2,472,416	Glycosyltransferase-like KOBITO 1	Os01g13200
	TraesCS1D02G007800	3,961,444–3,964,988	Ankyrin repeat family protein	Os01g01960
	TraesCS1D02G007900	3,968,895–3,969,443	MICOS complex subunit Mic25	Os05g01300
MQTL2A.2	TraesCS2A02G083000	38,218,064–38,220,520	Elongation factor 1-alpha	Os03g08010
	TraesCS2A02G083300	38,304,986–38,306,906	Elongation factor 1-alpha	Os03g08010
	TraesCS2A02G086400	39,704,402–39,709,256	AAA + ATPase domain	*OsRpt3*; *OSRPT2B*
	TraesCS2A02G087000	40,541,031–40,547,241	Adenosine/AMP deaminase domain	Os07g49270
	TraesCS2A02G088300	41,652,179–41,655,428	NmrA-like domain	Os12g16410
	TraesCS2A02G089300	42,470,945–42,476,145	Heat shock transcription factor	*OsHsfA2b*; *OsHSF5*
	TraesCS2A02G090000	43,133,651–43,137,076	AAA + ATPase domain	*OSRPT2B*
	TraesCS2A02G092200	45,085,317–45,085,622	Wound-induced protein WI12	Os03g18770
	TraesCS2A02G075800	33,696,041–33,701,785	DNA binding	Os04g19684
	TraesCS2A02G076700LC	38,490,262–38,490,831	Pol polyprotein	Os04g20220
	TraesCS2A02G076900	34,517,930–34,520,662	ER membrane protein complex subunit 8/9-like protein	Os04g20230
	TraesCS2A02G079500	36,047,811–36,053,052	Oxoglutarate dehydrogenase (succinyl-transferring) activity	Os07g49520
	TraesCS2A02G080000	36,138,685–36,141,909	LPS-induced tumor necrosis factor alpha factor	Os02g31100
	TraesCS2A02G082100	37,084,649–37,088,462	Peroxidase activity	*OsAPX1*; *OsAPXa*
	TraesCS2A02G075900	33,712,839–33,714,236	Leucine-rich repeat 2	*OsFbox194*
MQTL3D.1	TraesCS3D02G024500	8,285,414–8,287,617	Glyceraldehyde-3-phosphate dehydrogenase	Os01g02930
	TraesCS3D02G024700	8,336,528–8,341,354	Cytochrome P450	*OsCYP709C5*
	TraesCS3D02G026400	8,971,472–8,975,036	Fructose-bisphosphate aldolase class-I	Os11g07020
	TraesCS3D02G031900	11,747,403–11,752,024	WD40 repeat	*OsAIP1*
	TraesCS3D02G032000	11,755,792–11,762,962	Ubiquitin-conjugating enzyme E2	*OsUBC34*
MQTL4A.2	TraesCS4A02G472900LC	605,125,402–605,129,635	Putative S-adenosyl-L-methionine-dependent methyltransferase	Os01g62800
	TraesCS4A02G473000LC	605,128,287–605,128,517	S-adenosyl-L-methionine-dependent methyltransferases superfamily protein	NA
	TraesCS4A02G315500	605,656,378–605,659,792	Chaperonin Cpn60	Os12g17910
	TraesCS4A02G310700	603,377,077–603,380,232	Zinc finger C2H2-type	Os09g39660
MQTL4A.6	TraesCS4A02G442900	710,742,945–710,744,427	Peroxisomal biogenesis factor 11	Os06g03660
	TraesCS4A02G445300	713,352,055–713,352,438	Ozone-responsive stress-related protein	Os06g02420
MQTL6B.5	TraesCS6B02G772700LC	701,661,949–701,662,457	ATP-dependent 6-phosphofructokinase 1	NA
	TraesCS6B02G432600	701,871,210–701,874,404	Thiolase	*OsI57*
	TraesCS6B02G432700	701,886,743–701,890,627	Ribosomal protein L13	Os08g06474
	TraesCS6B02G432900	701,977,549–701,982,043	Aldo/keto reductase family	Os02g57240
	TraesCS6B02G433800	702,562,152–702,565,516	DHHC palmitoyltransferase	*OsPAT15*
	TraesCS6B02G434700	703,153,107–703,155,841	OTU-like cysteine protease	Os02g57410
	TraesCS6B02G436400	704,038,894–704,042,474	Serine-threonine protein phosphatase N-terminal domain	*OsPP41*
	TraesCS6B02G439300	704,879,300–704,881,854	PBS lyase HEAT-like repeat	Os12g43100
	TraesCS6B02G439400	704,882,414–704,885,861	Target SNARE coiled-coil homology domain	Os02g57510
	TraesCS6B02G783000LC	704,944,589–704,949,129	ATP binding	NA
	TraesCS6B02G439800	705,158,924–705,162,882	RING/U-box superfamily protein	Os11g18947
	TraesCS6B02G440000	705,282,693–705,285,851	B3 DNA binding domain	Os03g42230
	TraesCS6B02G440200	705,377,945–705,384,852	Metabolic process	Os06g19960
	TraesCS6B02G440500	705,497,185–705,500,263	Fibrillarin	Os02g57590
MQTL7B.1	TraesCS7B02G002900	1,203,205–1,208,405	COP1-interacting-like protein	*DEP2*; *EP2*; *SRS1*
	TraesCS7B02G005700	3,142,605–3,150,879	THIF-type NAD/FAD binding fold	Os02g30310
	TraesCS7B02G005800LC	2,014,362–2,018,322	NAC domain	Os01g18070
	TraesCS7B02G003000	1,254,814–1,262,214	COP1-interacting-like protein	*DEP2*; *EP2*; *SRS1*
	TraesCS7B02G003200	1,277,537–1,282,562	PB1 domain	Os07g25680
MQTL7B.3	TraesCS7B02G366700	630,552,409–630,552,871	Ubiquitin domain	Os06g46770
	TraesCS7B02G619400LC	632,490,144–632,492,343	GTPase activity	NA
	TraesCS7B02G377800	642,274,924–642,277,145	Ribosomal protein S8	Os02g15610
	TraesCS7B02G636000LC	644,027,912–644,028,247	Myosin-like protein XIG	NA
	TraesCS7B02G623100LC	634,562,940–634,564,127	F-box protein At5g41490	NA
	TraesCS7B02G370800	636,625,334–636,627,607	Ribosomal protein S13	Os03g58050
	TraesCS7B02G371900	637,769,054–637,774,747	RNA recognition motif domain	Os06g45910
	TraesCS7B02G372500	638,129,949–638,134,271	SANT/Myb domain	Os06g01670
	TraesCS7B02G372700	638,509,625–638,515,329	Conserved oligomeric Golgi complex subunit 7	Os06g45830
	TraesCS7B02G373000	638,882,526–638,885,183	Peptidase M41	*OsFtsH2*
MQTL7D.2	TraesCS7D02G148900	96,756,606–96,777,587	Chromatin-remodeling factor CHD3	*CHR702*
	TraesCS7D02G149000	97,615,140–97,617,186	SWEET sugar transporter	*OsSWEET15*
	TraesCS7D02G149300	98,292,586–98,293,620	Rtf2 RING-finger	Os06g08490
	TraesCS7D02G149500	98,408,253–98,411,417	DNA-directed RNA polymerase subunit beta	*DPL2*
	TraesCS7D02G149800	98,637,892–98,644,179	Ubiquitin carboxyl-terminal hydrolase	Os06g08530
	TraesCS7D02G150300	99,617,003–99,618,245	Thioredoxin-like fold	Os07g09310
	TraesCS7D02G150900	100,280,693–100,281,019	Proteolipid membrane potential modulator	*OsRCI2-8*
	TraesCS7D02G152400	101,084,476–101,087,850	Glutathione peroxidase	*OsGPX4*
	TraesCS7D02G152800	101,395,597–101,400,935	Serine-type carboxypeptidase activity	*OsSCP1*
	TraesCS7D02G153200	101,580,722–101,585,530	ATP-dependent DNA helicase	Os06g08740
	TraesCS7D02G154500	102,537,242–102,539,785	RNA-binding (RRM/RBD/RNP motifs) family protein	Os10g39510

## Discussions

Grain yield is influenced by the combination of kernel weight and number per spike [14, 49]. TKW is not only one of the critical components of grain yield, but also is commonly used as a common factor for determining commercial value in wheat. Kernel size and shape, including KL, KW and KT, are strongly and positively correlated with TKW [50–52]. A bigger kernel positively affects wheat kernel weight, yield and commercial value [40, 53]. Kernel size-related traits influence wheat yield by regulating TKW, and both are associated with high heritability [16, 54–59].

We observed significant and positive correlations between KL, KW and KDR (*r* = 0.45, *P* < 0.01 and *r* = 0.71, *P* < 0.01, respectively), KW and KT (*r* = 0.41, *P* < 0.01). Meanwhile, a negative and significant correlation was also observed between KT and KDR (*r* = -0.42, *P* < 0.01) (Fig. 2), which is consistent with previous studies [13, 50, 60, 61]. It is known that KL reached its maximum value 15 days after anthesis, while KW and KT reached their maximum value four weeks after anthesis [62, 63]. KL showed the highest heritability (0.89) in this study, followed by KDR (0.70), KW (0.67) and KT (0.61) (Table S2), which is consistent with previous studies [7, 62–64]. Therefore, increasing KL and KW through genetic improvement has a positive effect on the grain weight and yield of wheat.

Many QTLs and genes for kernel size have been identified on 21 chromosomes in wheat [16, 61, 64–67, 69, 70]. In this study, 32 QTLs for KL, KW, KDR and KT were found on 17 chromosomes (Table S3, Fig. 3). Of these, a stable QTL, *QKW.acs-1A*, identified in E4, E6 and E7, is mapped in the *Xcfa2219-Xgwm99* interval on chromosome 1A only under DS environments. Li et al. (2012) identified a major QTL with a PVE of 40.79% that shares the same flanking marker *Xgwm99* with *QKW.acs-1A* [71]. In addition, the stable QTLs *QKL.acs-1A* and *QKW.acs-1A* share the same flanking marker *Xwmc99* with the QTLs *QGw.ccsu-1A.3* reported by Mir et al. (2012) [72]. This suggests that the marker *Xgwm99* can be used for marker-assisted selection in wheat breeding programs. *QKL.acs-2D*, located in the interval of *Xgwm157-Xwmc41*, shared a common flanking marker (*Xwmc41*) with *QTKW.ncl-2D.2* [54]. *QKL.acs-2D*, located in the *Xgwm157-Xwmc41* interval on chromosome 2D, strongly overlapped with the different environmental QTLs for KDR (*QKDR.acs-2D*) and KW (*QKW.acs-2D*). In addition, *QKL.acs-6B* was identified in E1, E2, E3, E6 and E7, with a PVE ranging from 4.07% to 8.26%. This indicates that kernel size-related traits are closely linked and represent one of the crucial elements in the regulation of kernel weight.

MQTL analysis is a powerful strategy for validating consistent QTLs by integrating independent QTLs from different trials on a consensus or reference map [27, 73]. In the present study, a total of 1103 initial QTLs from previous mapping studies and identified in this study were performed MQTL analysis to identify key genomic regions linked to kernel size-related traits in wheat (Table S4, Fig. 4). As a result, 346 initial QTLs were finally refined into 58 MQTLs on chromosomes 1B, 1D, 2A, 3D, 4A, 5B, 5D, 6B, 7A, 7B and 7D (Table S5, Fig. 5). The average 95% CI of MQTLs (4.46 cM) was 3.26-fold less than that of initial QTLs (14.54 cM). The result was similar to previous MQTL analysis for grain yield and yield-related traits, where the average CI of MQTLs was 2.9-fold lower than that of the initial QTLs [37]. Most of the MQTLs in the present study controlled more than one trait, likely indicating either a tight linkage of genes or the presence of pleiotropic genes for controlling kernel size-related traits [37, 43, 48, 73]. By the peak marker sequences compared with the wheat genome reference sequence of Chinese Spring, 58 MQTLs had definite physical positions and the physical intervals ranged from 1.54 Kb to 580.66 Mb (Table S5). Of these, six MQTLs, such as *MQTL1D.2*, *MQTL4A.2*, *MQTL7B.1*, *MQTL7B.4*, *MQTL7B.5* and *MQTL7B.6*, showed narrower physical intervals (< 5 Mb), shorter genetic distance (< 10 cM) and more initial QTLs (> 2) (Table S5). These MQTLs are promised to be used in future marker-assisted selection for improving kernel size, and for isolating key genes by the map-based cloning approach in wheat.

Candidate genes related to important agronomic traits in wheat have been identified by MQTL analysis [37, 40, 43, 74, 75]. Nadolska-Orczyk et al. (2017) classified the genes controlling kernel yield into five categories: transcription factors, growth regulator signaling, carbohydrate metabolism, cell division and proliferation and flowering regulators [76]. Understanding the genetic and physiological pathways involved in grain development is of great help for investigating traits related to kernel size. In this study, we detected 1864 potential candidate genes in 12 core MQTL intervals with a physical interval of less than 20 Mb using the wheat genome reference sequence of Chinese Spring. Among 1864 potential candidate genes, 70 candidate genes were mainly expressed in the spike and grain at different developmental stages (Table 1, Fig. 9), consistent with those previously reported by Yang et al. (2021) [37]. In recent years, the analysis of homology relationships between wheat and rice facilitates the cloning of several yield-related genes such as *TaFlo-A1* [77], *TaCKX6-D1* [78] and *TaTGW6-A1* [79]. In the present study, 17 out of 70 candidate genes homologous to rice genes were found within nine core MQTL intervals (Table 1). Of these, a key gene *TraesCS3D02G024700* in the *MQTL3D.1* interval was homologous to the gene *OsCYP709C5* involved in regulating cytochrome P450 in rice [80]. Guo et al. (2021) also showed that constitutive overexpression of *TaCYP78A5* significantly increased seed size and weight [81]. The ubiquitin–proteasome pathway has been associated with seed size development in wheat and rice. The corresponding genes, *e.g.*, *TaGW2-6A/6B* [82, 83] and *OsUBC* [84] have been cloned in wheat and rice, respectively. According to a previous study, carbohydrate metabolism is essential to yield and yield-related traits [76]. The gene *TraesCS7D02G149000* identified in the *MQTL7D.2* region was homologous to the genes of *OsSWEET15* in rice [85] and *TaSWEETs* in wheat [86, 87], which were identified as the key gene involved in the sucrose transport pathway in rice [85] and floral development in wheat [76]. The gene of *TraesCS7D02G149500* (MQTL7D.2) was identified as an orthologous gene of *DPL1/2*, involved in pollen hybrid incompatibility in rice [88]. In this study, the orthologous genes of *DEP2*, *EP2* and *SRS1* were found in the *MQTL7B.1* region as *TraesCS7B02G002900* and *TraesCS7B02G003000*, which was involved in regulating kernel size and yield [89, 90]. In addition, the remaining 53 candidate genes were involved in various signaling pathways, such as zinc finger protein [91], transcription factors [17] and glycosyltransferase [92], which are also involved in the regulation of yield and yield-related traits.

## Conclusions

In this study, we found that kernel size-related traits in wheat are predominantly regulated by genetic factors with moderate and high heritability. Most of stable QTLs were detected under both well-watered and drought-stressed conditions. Potential candidate genes expressed in spike and grain were identified through meta-QTL and *in-silico* expression analysis. The markers closely linked to stable QTLs had great potential in the marker-assisted breeding program and the identification of candidate genes advanced the understanding of the genetic basis governing kernel size in wheat.

## Methods

### Plant materials and field trials

A RILs population consisting of 120 lines derived from the cross between two winter wheat cultivars, Longjian19 and Q9086 [93]. The male parent, Longjian19, released by the Gansu Academy of Agricultural Sciences, Lanzhou, Gansu, is an elite drought-tolerant variety widely grown in rainfed areas (300–500 mm annual rainfall) in northwest China. The female parent Q9086, is a high-yielding cultivar developed by Northwest Agriculture and Forestry University, Yangling, Shanxi, China. It is suitable for cultivation under conditions with sufficient water and high fertility. The two parents differ significantly from several physiological and agronomical traits, especially under rainfed environments [93–95].

Field trials were conducted at Yuzhong farm station, Gansu, China (35°48'N, 104°18'E, altitude 1860 m) during the growing seasons in 2015–2016 under drought-stressed (DS, designated E1) and well-watered conditions (WW, designated E2), while in 2016–2017 only under drought-stressed conditions (designated E3). Field trials were also conducted at Tongwei farm station, Gansu, China (35°11′N, 105°19′E, altitude 1750 m) during the 2017–2018, 2018–2019, and 2019–2020 growing seasons. Planting in 2017–2018 was conducted under drought-stressed (designated E4) and well-watered conditions (designated E5), while the 2018–2019 and 2019–2020 cropping seasons were conducted under drought-stressed conditions only (designated E6 and E7, respectively). The two cropping sites are characterized by a typical dry inland environmental condition in Northwest China, where the annual average temperature is about 7.0 °C, the annual rainfall is less than 400 mm with approximately 60% falling from July to September, but the annual evapotranspiration capacity is more than 1500 mm. The two water treatments in different locations and years were conducted in field conditions without any rainout shelter. The DS treatments were equivalent to the rainfed condition in each growing season, whereas the WW treatments were irrigated with a water supply of 75 mm at the spike emergence (Zadoks 55) and grain filling (Zadoks 71) stages, respectively. Here, the decimal codes for the growth stages of wheat are described by Zadoks et al. (1974) [96]. In this case, the rainfall of the DS plots in each field environment was 164.3 mm (E1) to 296.5 mm (E7) (Fig. S1). All progenies and parents were sown in late September and harvested in early July of the following year. A randomized complete block design (RCBD) was conducted with three replications for each line and parent. Each plot consisted of six 1 m rows, 0.2 m spacing, with a sowing rate of 60 seeds per row. Field management followed local wheat cultivation practices.

After harvesting, two hundred seeds for each line were used to measure kernel length (KL), kernel width (KW) and kernel diameter ratio (KDR) with the SC-G wheat grain appearance quality image analysis system (Hangzhou WSeen Detection Technology Co., Ltd, Hangzhou, China). The kernel thickness (KT) was determined with a vernier caliper. All measurements were conducted with three biological replicates. The average values of the traits were used for QTL analysis.

### Statistical analysis

Statistical analysis and analysis of variance (ANOVA) were performed using SPSS 22.0 (IBM Corporation, Armonk, NY, United States). According to the method described by Toker et al. (2004) [97], the broad-sense heritability (*h*^*2*^) was estimated across environments using the formula:

$${h}^{2}={\sigma }_{g}^{2}/\left({\sigma }_{g}^{2}+{\sigma }_{ge}^{2}/r+{\sigma }_{e}^{2}/re\right)$$

where ${\sigma }_{g}^{2}$, ${\sigma }_{ge}^{2}$ and ${\sigma }_{e}^{2}$ estimate genotype, genotype × environment interaction and residual error variances, respectively, and *e* and *r* are the numbers of environments and replicates per environment, respectively. The correlation among KL, KW, KDR and KT in the RILs population was also assessed.

### Construction of linkage map and QTL analysis

For QTL mapping, a genetic map consisting of 524 SSR markers, described in a previous study was used [98]. These markers were distributed among 21 linkage groups and covered a total genetic distance of 2266.72 cM with an average distance of 4.33 cM between adjacent markers.

The inclusive composite interval mapping (ICIM) method was performed using the QTL software IciMapping V4.1 to determine the positions and effects of QTLs [99]. QTL with LOD value ≥ 2.5, as determined by 1000 permutation tests at *P* ≤ 0.05, were declared for the presence of significant QTL. QTLs were named based on the International Rules for Genetic Nomenclature (http://wheat.pw.usda.gov/ggpages/wgc/98/intro.htm). QTLs detected in at least three of seven environments were considered stable QTLs. QTLs for a trait identified with common flanking markers or overlapping CIs were treated as one QTL, with the CI reassigned by overlapping genetic positions.

### Initial QTL collections used for MQTL analysis

A total of 1071 QTLs for KL, KW, KDR and KT traits derived from 36 bi-parental populations were retrieved from 34 published studies from 2007 to 2020 (Table S4). The size of the mapping populations varied from 99 to 547 lines of different types, including three double haploid (DH), seven F_2_ and 26 RILs populations evaluated in different years and locations. The population information, including target traits, population parents, population types, and the number of markers used in the genetic map, was listed in Table S4.

### QTLs localization on the reference map

A high-density map containing 7352 markers, including SSR, DArT, SNP and other types of markers, was used as a reference map in this study [75]. The total length of the reference map is 4994.0 cM with an average distance of 0.68 cM. The original QTL data and associated individual genetic maps from previous studies, and the reference map, were used as input files to create a consensus map (Fig. S2) and perform MQTL analysis with BioMercator V4.2.3 [100].

The position, chromosome groups, proportion of phenotypic variance explained (PVE or R^2^), and logarithm of odds ratio (LOD score) were recorded for each of the QTLs in the 36 studies. The formula CI = 530/(N × R^2^) for BC and F_2_ lines, CI = 287/(N × R^2^) for DH lines, and CI = 163/(N × R^2^) for RILs lines was applied to calculate the 95% CIs of QTLs, where N is the population size and R^2^ is the proportion of phenotypic variance explained of the QTL [101]. For QTLs without well-defined LOD scores and R^2^, these criteria were arbitrarily set at 3 and 10%, respectively. All collected QTLs with appropriate information were projected onto the reference map using BioMercator V4.2.3 [100]. The approach proposed by Goffinet and Gerber (2000) [27] was used when the number of QTLs per chromosome was ten or less, while the two-step algorithm was used when the number of QTLs per chromosome was higher than ten [102]. The Akaike Information Criterion (AIC) statistics were used to determine the best model for defining the number of MQTLs or "true" QTLs that best represent the original QTLs. The algorithms and statistical procedures implemented in this software are well described in previous studies [100, 102, 103].

### Identification of candidate genes

To identify candidate genes, initially, the marker or its related primer sequences on both sides of the MQTL confidence intervals were manually searched using URGI Wheat (https://wheat-urgi.versailles.inra.fr), GrainGenes (https://wheat.pw.usda.gov/GG3/), DArT (https://www.diversityarrays.com) and the Illumina company (https://www.illumina.com) databases. The obtained sequences were then aligned to IWGSC RefSeq v1.1 (https://wheat-urgi.versailles.inra.fr/) to find the physical location of each marker. Candidate genes for this MQTL with a physical interval of less than 20 Mb were identified, and their associated functions were compared to choose the best possible candidates. The candidate genes were also investigated using Gene Ontology (GO) and Kyoto Encyclopedia of Genes and Genomes **(**KEGG) enrichment analyses using Omicshare online tools (https://www.omicshare.com/).

### *In-silico* expression analysis of candidate genes

The transcriptomic data of several wheat tissues deposited in the Expression Visualization and Integration Platform (expVIP, https://www.wheat-expression.com/) were downloaded to study the *in-silico* tissue expression of candidate genes [104]. This included 18 tissues throughout the wheat growth period [105]. The expression levels of candidate genes were assessed by transcripts per million (TPM) and visualized using the heatmap of TBtools software (https://github.com/CJChen/TBtools/releases).

## Supplementary information

**Additional file 1: Fig. S1.** The rainfall records (mm) for each growing season in seven tested environments. E1-E3 are the experimental environments in Yuzhong farm station during 2015-2016 under DS and WW conditions and during 2016-2017 under DS conditions, respectively. E4-E7 are the experimental environments in Tongwei farm station during 2017-2018 under DS and WW conditions and 2018-2020 under DS conditions, respectively. **Fig. S2.**Distribution of the markers on the consensus map used for MQTL analysis in thisstudy.

**Additional file 2: Table S1.**  Evaluation of the kernel size-related traits in RILs population and their parents under different environments. **Table S2.** ANOVA and heritability of kernel size-related traits in the Q9086/Longjian19 RILs population. **Table S3.** Summary of the QTLs identified for kernel size-related traits in all the environments in the Q9086/Longjian19 RILs population. **Table S4.**  Summary of the QTL studies used for conducting MQTL analysis for kernel-size related traits in wheat. **Table S5.** MQTLs for kernel size-related traits identified in this study. **Table S6.** The information of candidate genes predicted within seven key intervals for stable QTLs and QTL clusters underlying kernel traits 

## Data Availability

All data generated or analyzed during this study are included in this published article and its supplementary information files. The datasets used and/or analyzed during the current study are available from the corresponding author on reasonable request. The original QTL mapping datasets presented in this study can be found in online repositories.

## Abbreviations

AIC: Akaike Information Criterion
ANOVA: Analysis of variance
BC: Back cross
CG: Candidate genes
CI: Confidence interval
cM: Centimorgan
DH: Double haploid
DS: Drought-stressed
GO: Gene Ontology
GWAS: Genome-wide association study
*h*^*2*^: Broad-sense heritability
ICIM: Inclusive composite interval mapping
KDR: Kernel diameter ratio
KEGG: Kyoto Encyclopedia of Genes and Genomes
KL: Kernel length
KNS: Number of kernel per spike
KT: Kernel thickness
KW: Kernel width
LOD: Logarithm of odds ratio
MQTL: Meta-QTL
PVE: Phenotypic variation explained
QTL: Quantitative trait loci
RCBD: Randomized complete block design
RIL: Recombinant inbred lines
SN: Spike number per unit area
SNP: Single nucleotide polymorphic
SSR: Simple sequence repeat
TKW: Thousand kernel weight
TPM: Transcripts per million
WW: Well-watered

## References

[1] Food and Agriculture Organization of the United Nations. https://www.fao.org/faostat/en/.

[2] Langridge P (2013). Wheat genomics and the ambitious targets for future wheat production. Genome.

[3] Hawkesford MJ, Araus JL, Park R, Calderini D, Miralles D, Shen T, Zhang J, Parry AJ (2013). Prospects of doubling global wheat yields. Food and Energy Security.

[4] Kesavan M, Song JT, Seo HS (2013). Seed size: A priority trait in cereal crops. Physiol Plant.

[5] Sehgal D, Mondal S, Guzman C, Garcia Barrios G, Franco C, Singh R, Dreisigacker S (2019). Validation of Candidate Gene-Based Markers and Identification of Novel Loci for Thousand-Grain Weight in Spring Bread Wheat. Front Plant Sci.

[6] Dholakia BB, Ammiraju SS, Singh H, Lagu MD, Röder MS, Rao VS, Dhaliwal HS, Ranjekar PK, Gupta VS (2003). Molecular marker analysis of kernel size and shape in bread wheat. Plant Breeding.

[7] Gegas VC, Nazari A, Griffiths S, Simmonds J, Fish L, Orford S, Sayers L, Doonan JH, Snape JW (2010). A genetic framework for grain size and shape variation in wheat. Plant Cell.

[8] Wang S, Li S, Liu Q, Wu K, Zhang J, Wang S, Wang Y, Chen X, Zhang Y, Gao C, Wang F, Huang H, Fu X (2015). The *OsSPL16* - *GW7* regulatory module determines grain shape and simultaneously improves rice yield and grain quality. Nat Genet.

[9] Williams K, Sorrells ME. Three-dimensional seed size and shape QTL in hexaploid wheat (*Triticum aestivum* L.) populations. Crop Science. 2014; 54(1): 98–110. 10.2135/cropsci2012.10.0609.

[10] Kumari S, Jaiswal V, Mishra VK, Paliwal R, Balyan HS, Gupta PK. QTL mapping for some grain traits in bread wheat (*Triticum aestivum* L.). Physiology and Molecular Biology of Plants. 2018; 24(5): 909–920. 10.1007/s12298-018-0552-1. 10.1007/s12298-018-0552-1 PMC6103944 30150865

[11] Hu J, Wang X, Zhang G, Jiang P, Chen W, Hao Y, Ma X, Xu S, Jia J, Kong L, Wang H (2020). QTL mapping for yield-related traits in wheat based on four RIL populations. Theor Appl Genet.

[12] Cao S, Xu D, Hanif M, Xia X, He ZH (2020). Genetic architecture underpinning yield component traits in wheat. Theor Appl Genet.

[13] Sun XY, Wu K, Zhao Y, Kong FM, Han GZ, Jiang HM, Huang XJ, Li RJ, Wang HG, Li SS (2009). QTL analysis of kernel shape and weight using recombinant inbred lines in wheat. Euphytica.

[14] Tsilo TJ, Hareland GA, Simsek S, Chao S, Anderson JA (2010). Genome mapping of kernel characteristics in hard red spring wheat breeding lines. Theor Appl Genet.

[15] Prashant R, Kadoo N, Desale C, Kore P, Dhaliwal HS, Chhuneja P, Gupta V. Kernel morphometric traits in hexaploid wheat (*Triticum aestivum* L.) are modulated by intricate QTL × QTL and genotype × environment interactions. Journal of Cereal Science. 2012; 56(2): 432–439. 10.1016/j.jcs.2012.05.010.

[16] Kumar A, Mantovani EE, Seetan R, Soltani A, Echeverry-Solarte M, Jain S, Simsek S, Doehlert D, Alamri MS, Elias EM, Kianian SF, Mergoum M (2016). Dissection of Genetic Factors underlying Wheat Kernel Shape and Size in an Elite × Nonadapted Cross using a High Density SNP Linkage Map. The Plant Genome.

[17] Brinton J, Simmonds J, Uauy C (2018). Ubiquitin-related genes are differentially expressed in isogenic lines contrasting for pericarp cell size and grain weight in hexaploid wheat. BMC Plant Biol.

[18] Yan X, Zhao L, Ren Y, Dong Z, Cui D, Chen F (2019). Genome-wide association study revealed that the *TaGW8* gene was associated with kernel size in Chinese bread wheat. Sci Rep.

[19] Muqaddasi QH, Brassac J, Ebmeyer E, Kollers S, Korzun V, Argillier O, Stiewe G, Plieske J, Ganal MW, Röder MS (2020). Prospects of GWAS and predictive breeding for European winter wheat’s grain protein content, grain starch content, and grain hardness. Sci Rep.

[20] Gahlaut V, Jaiswal V, Balyan HS, Joshi AK, Gupta PK. Multi-Locus GWAS for Grain Weight-Related Traits Under Rain-Fed Conditions in Common Wheat (*Triticum aestivum* L.). Frontiers in Plant Science. 2021; 12: 1–13. 10.3389/fpls.2021.758631.10.3389/fpls.2021.758631 PMC8568012 34745191

[21] Gao L, Meng C, Yi T, Xu K, Cao H, Zhang S, Yang X, Zhao Y (2021). Genome-wide association study reveals the genetic basis of yield- and quality-related traits in wheat. BMC Plant Biol.

[22] Malik P, Kumar J, Sharma S, Meher PK, Balyan HS, Gupta PK, Sharma S (2022). GWAS for main effects and epistatic interactions for grain morphology traits in wheat. Physiol Mol Biol Plants.

[23] Tong J, Zhao C, Sun M, Fu L, Song J, Liu D, Zhang Y, Zheng J, Pu Z, Liu L, Rasheed A, Li M, Xia X, He Z, Hao Y. High Resolution Genome Wide Association Studies Reveal Rich Genetic Architectures of Grain Zinc and Iron in Common Wheat (*Triticum aestivum* L.). Frontiers in Plant Science. 2022; 13: 758631–758644. 10.3389/fpls.2022.840614.10.3389/fpls.2022.840614 PMC8966881 35371186

[24] Simmonds J, Scott P, Leverington-Waite M, Turner AS, Brinton J, Korzun V, Snape J, Uauy C. Identification and independent validation of a stable yield and thousand grain weight QTL on chromosome 6A of hexaploid wheat (*Triticum aestivum* L.). BMC Plant Biology. 2014; 14(1): 191–204. 10.1186/s12870-014-0191-9.10.1186/s12870-014-0191-9 PMC4105860 25034643

[25] Guan P, Shen X, Mu Q, Wang Y, Wang X, Chen Y, Zhao Y, Chen X, Zhao A, Mao W, Guo Y, Xin M, Hu Z, Yao Y, Ni Z, Sun Q, Peng H. Dissection and validation of a QTL cluster linked to Rht-B1 locus controlling grain weight in common wheat (*Triticum aestivum* L.) using near-isogenic lines. Theoretical and Applied Genetics. 2020; 133(9): 2639–2653. 10.1007/s00122-020-03622-z.10.1007/s00122-020-03622-z 32488301

[26] Khahani B, Tavakol E, Shariati V, Fornara F (2020). Genome wide screening and comparative genome analysis for Meta-QTLs, ortho-MQTLs and candidate genes controlling yield and yield-related traits in rice. BMC Genomics.

[27] Goffinet B, Gerber S (2000). Quantitative trait loci: A meta-analysis. Genetics.

[28] Coque M, Martin A, Veyrieras JB, Hirel B, Gallais A. Genetic variation for N-remobilization and postsilking N-uptake in a set of maize recombinant inbred lines. 3. QTL detection and coincidences. Theoretical and Applied Genetics. 2008; 117(5): 729–747. 10.1007/s00122-008-0815-2.10.1007/s00122-008-0815-2 18566796

[29] Truntzler M, Barrière Y, Sawkins MC, Lespinasse D, Betran J, Charcosset A, Moreau L (2010). Meta-analysis of QTL involved in silage quality of maize and comparison with the position of candidate genes. Theor Appl Genet.

[30] Chen L, An Y, Li YX, Li C, Shi Y, Song Y, Zhang D, Wang T, Li Y (2017). Candidate loci for yield-related traits in maize revealed by a combination of metaQTL analysis and regional association mapping. Front Plant Sci.

[31] Guo J, Chen L, Li Y, Shi Y, Song Y, Zhang D, Li Y, Wang T, Yang D, Li C (2018). Meta-QTL analysis and identification of candidate genes related to root traits in maize. Euphytica.

[32] Ballini E, More JB, Droc G, Price A, Courtois B, Notteghem JL, Tharreau D (2008). A genome-wide meta-analysis of rice blast resistance genes and quantitative trait loci provides new insights into partial and complete resistance. Mol Plant Microbe Interact.

[33] Islam MS, Ontoy J, Subudhi PK. Meta-analysis of quantitative trait loci associated with seedling-stage salt tolerance in rice (*Oryza Sativa* L.). Plants. 2019; 8(2): 4–13. 10.3390/plants8020033.10.3390/plants8020033 PMC6409918 30699967

[34] Sun YN, Pan JB, Shi XL, Du XY, Wu Q, Qi ZM, Jiang HW, Xin DW, Liu CY, Hu GH, Chen QS (2012). Multi-environment mapping and meta-analysis of 100-seed weight in soybean. Mol Biol Rep.

[35] Saini DK, Srivastava P, Pal N. Meta-QTLs, ortho-meta-QTLs and candidate genes for grain yield and associated traits in wheat (*Triticum aestivum* L.). Theor Appl Genet. 2022; 135: 1049–1081. 10.1007/s00122-021-04018-3. 10.1007/s00122-021-04018-3 34985537

[36] Saini DK, Chahal A, Pal N. Meta-analysis reveals consensus genomic regions associated with multiple disease resistance in wheat (*Triticum aestivum* L.). Mol Breeding. 2022; 42: 1–23. 10.1007/s11032-022-01282-z. 10.1007/s11032-022-01282-z PMC10248701 37309411

[37] Yang Y, Amo A, Wei D, Chai Y, Zheng J, Qiao P, Cui C, Lu S, Chen L, Hu YG (2021). Large-scale integration of meta-QTL and genome-wide association study discovers the genomic regions and candidate genes for yield and yield-related traits in bread wheat. Theor Appl Genet.

[38] Zhang LY, Liu DC, Guo XL, Yang WL, Sun JZ, Wang DW, Zhang A (2010). Genomic distribution of quantitative trait loci for yield and yield-related traits in common wheat. J Integr Plant Biol.

[39] Tyagi S, Mir RR, Balyan HS. Interval mapping and meta-QTL analysis of grain traits in common wheat (*Triticum aestivum* L.). Euphytica. 2015; 201(3): 367–380. 10.1007/s10681-014-1217-y.

[40] Liu H, Mullan D, Zhang C, Zhao S, Li X, Zhang A, Lu Z, Wang Y, Yan G (2020). Major genomic regions responsible for wheat yield and its components as revealed by meta-QTL and genotype–phenotype association analyses. Planta.

[41] Soriano JM, Royo C (2015). Dissecting the genetic architecture of leaf rust resistance in wheat by QTL meta-analysis. Phytopathology.

[42] Acuña-Galindo MA, Mason RE, Subramanian NK, Hays DB (2015). Meta-analysis of wheat QTL regions associated with adaptation to drought and heat stress. Crop Sci.

[43] Kumar A, Saripalli G, Jan I, Kumar K, Sharma PK, Balyan HS, Gupta PK. Meta-QTL analysis and identification of candidate genes for drought tolerance in bread wheat (*Triticum aestivum* L.). Physiology and Molecular Biology of Plants. 2020; 26(8): 1713–1725. 10.1007/s12298-020-00847-6.10.1007/s12298-020-00847-6 PMC7415061 32801498

[44] Soriano JM, Colasuonno P, Marcotuli I, Gadaleta A (2021). Meta-QTL analysis and identification of candidate genes for quality, abiotic and biotic stress in durum wheat. Sci Rep.

[45] Pal N, Saini DK, Kumar S. Meta-QTLs, ortho-MQTLs and candidate genes for the traits contributing to salinity stress tolerance in common wheat (*Triticum aestivum* L.). Physiology and Molecular Biology of Plants. 2021; 27: 2767–2786. 10.1007/s12298-021-01112-0.10.1007/s12298-021-01112-0 PMC8720133 35035135

[46] Liu Y, Salsman E, Wang R, Galagedara N, Zhang Q, Fiedler JD, Liu Z, Xu S, Faris JD, Li X (2020). Meta-QTL analysis of tan spot resistance in wheat. Theor Appl Genet.

[47] Amo A, Soriano JM (2021). Unravelling consensus genomic regions conferring leaf rust resistance in wheat via meta-QTL analysis. The Plant Genome.

[48] Jan I, Saripalli G, Kumar K, Kumar A, Singh R, Batra R, Sharma PK, Balyan HS, Gupta PK (2021). Meta-QTLs and candidate genes for stripe rust resistance in wheat. Sci Rep.

[49] Neuweiler JE, Maurer HP, Würschum T (2020). Long-term trends and genetic architecture of seed characteristics, grain yield and correlated agronomic traits in triticale (×*Triticosecale Wittmack*). Plant Breeding.

[50] Xiao Y, He S, Yan J, Zhang Y, Zhang Y, Wu Y, Xia X, Tian J, Ji W, He Z. Molecular mapping of quantitative trait loci for kernel morphology traits in a non-1BL.1RS1BL.1RS wheat cross. Crop and Pasture Science. 2011; 62(8): 625–638. 10.1071/CP11037.

[51] Griffiths S, Wingen L, Pietragalla J, Garcia G, Hasan A, Miralles D, Calderini DF, Ankleshwaria JB, Waite ML, Simmonds J, Snape J, Reynolds M (2015). Genetic dissection of grain size and grain number trade-offs in CIMMYT wheat germplasm. PLoS ONE.

[52] Zhang X, Larson SR, Gao L, Teh SL, DeHaan LR, Fraser M, Sallam A, Kantarski T, Frels K, Poland J, Wyse D, Anderson JA (2017). Uncovering the Genetic Architecture of Seed Weight and Size in Intermediate Wheatgrass through Linkage and Association Mapping. The Plant Genome.

[53] Cui F, Ding A, Li J, Zhao C, Li X, Feng D, Wang X, Wang L, Gao J, Wang H (2011). Wheat kernel dimensions: How do they contribute to kernel weight at an individual QTL level?. J Genet.

[54] Ramya P, Chaubal A, Kulkarni K, Gupta L, Kadoo N, Dhaliwal HS, Chhuneja P, Lagu M, Gupta V. QTL mapping of 1000-kernel weight, kernel length, and kernel width in bread wheat (*Triticum aestivum* L.). Journal of Applied Genetics. 2010; 51(4): 421–429. 10.1007/BF03208872.10.1007/BF03208872 21063060

[55] Hasan AK, Herrera J, Lizana C, Calderini DF (2011). Carpel weight, grain length and stabilized grain water content are physiological drivers of grain weight determination of wheat. Field Crop Res.

[56] Ma Y, Chen G, Zhang L, Liu Y, Liu D, Wang J, Pu Z, Zhang L, Lan X, Wei Y, Liu C, Zheng Y. QTL Mapping for Important Agronomic Traits in Synthetic Hexaploid Wheat Derived from Aegiliops tauschii ssp. tauschii. Journal of Integrative Agriculture. 2014; 13: 1835–1844. 10.1016/S2095-3119(13)60655-3.

[57] Li M, Wang Z, Shen W, Sun F, Xi Y, Liu S (2015). Quantitative trait loci analysis for kernel-related characteristics in common wheat (*Triticum aestivum* L.). Crop Science.

[58] Qu X, Liu J, Xie X, Xu Q, Tang H, Mu Y, Pu Z, Li Y, Ma J, Gao Y, Jiang Q, Liu Y, Chen G, Wang J, Qi P, Habib A, Wei Y, Zheng Y, Lan X, Ma J. Genetic Mapping and Validation of Loci for Kernel-Related Traits in Wheat (*Triticum aestivum* L.). Frontiers in Plant Science. 2021; 12: 1–17. 10.3389/fpls.2021.667493.10.3389/fpls.2021.667493 PMC8215603 34163507

[59] Schierenbeck M, Alqudah AM, Lohwasser U, Tarawneh RA, Simón MR, Börner A (2021). Genetic dissection of grain architecture-related traits in a winter wheat population. BMC Plant Biol.

[60] Cui F, Zhao C, Ding A, Li J, Wang L, Li X, Bao Y, Li J, Wang H (2014). Construction of an integrative linkage map and QTL mapping of grain yield-related traits using three related wheat RIL populations. Theor Appl Genet.

[61] Wu QH, Chen YX, Zhou SH, Fu L, Chen JJ, Xiao Y, Zhang D, Ouyang SH, Zhao XJ, Cui Y, Zhang DY, Liang Y, Wang ZZ, Xie JZ, Qin JX, Wang GX, Li DL, Huang YL, Yu MH, Liu ZY (2015). High-density genetic linkage map construction and QTL mapping of grain shape and size in the wheat population Yanda 1817 x Beinong6. PLoS ONE.

[62] Lizana XC, Riegel R, Gomez LD, Herrera J, Isla A, McQueen-Mason SJ, Calderini, DF. Expansins expression is associated with grain size dynamics in wheat (*Triticum aestivum* L.). Journal of Experimental Botany. 2010; 61(4): 1147–1157. 10.1093/jxb/erp380.10.1093/jxb/erp380 PMC2826655 20080826

[63] Xie Q, Mayes S, Sparkes DL (2015). Carpel size, grain filling, and morphology determine individual grain weight in wheat. J Exp Bot.

[64] Breseghello F, Sorrells ME (2007). QTL analysis of kernel size and shape in two hexaploid wheat mapping populations. Field Crop Res.

[65] Williams K, Munkvold J, Sorrells M. Comparison of digital image analysis using elliptic Fourier descriptors and major dimensions to phenotype seed shape in hexaploid wheat (*Triticum aestivum* L.). Euphytica. 2013; 190(1): 99–116. 10.1007/s10681-012-0783-0.

[66] Okamoto Y, Nguyen AT, Yoshioka M, Iehisa M, Takumi S (2013). Identification of quantitative trait loci controlling grain size and shape in the D genome of synthetic hexaploid wheat lines. Breed Sci.

[67] Huang Y, Kong Z, Wu X, Cheng R, Yu D, Ma Z (2015). Characterization of three wheat grain weight QTLs that differentially affect kernel dimensions. Theor Appl Genet.

[68] Bhusal N, Sarial AK, Sharma P, Sareen S (2017). Mapping QTLs for grain yield components in wheat under heat stress. PLoS ONE.

[69] Desiderio F, Zarei L, Licciardello S, Cheghamirza K, Farshadfar E, Virzi N, Sciacca F, Bagnaresi P, Battaglia R, Guerra D, Palumbo M, Cattivelli L, Mazzucotelli E (2019). Genomic regions from an iranian landrace increase kernel size in durum wheat. Front Plant Sci.

[70] Xin F, Zhu T, Wei S, Han Y, Zhao Y, Zhang D, Ma L, Ding Q (2020). QTL Mapping of Kernel Traits and Validation of a Major QTL for Kernel Length-Width Ratio Using SNP and Bulked Segregant Analysis in Wheat. Sci Rep.

[71] Li M, Yang R, Li Y, Cui G, Wang Z, Xi Y, Liu S. QTL analysis of kernel characteristics using a recombinant inbred lines (RILs) population derived from the cross of *Triticum polonicum* L. and *Triticum aestivum* L. line "Zhong 13". Journal of Triticeae Crops. 2012; 32: 813–819.

[72] Mir RR, Kumar N, Jaiswal V, Girdharwal N, Prasad M, Balyan HS, Gupta PK (2012). Genetic dissection of grain weight in bread wheat through quantitative trait locus interval and association mapping. Mol Breeding.

[73] Saini DK, Chopra Y, Pal N, Chahal A, Srivastava P, Gupta PK. Meta-QTLs, ortho-MQTLs and candidate genes for nitrogen use efficiency and root system architecture in bread wheat (*Triticum aestivum* L.). Physiology and Molecular Biology of Plants. 2021; 27: 2245–2267. 10.1007/s12298-021-01085-0.10.1007/s12298-021-01085-0 PMC8526679 34744364

[74] Quraishi UM, Pont C, Ain QU, Flores R, Burlot L, Alaux M, Quesneville H, Salse J. Combined genomic and genetic data integration of major agronomical traits in bread wheat (*Triticum aestivum* L.). Frontiers in Plant Science. 2017; 8: 1843–1852. 10.3389/fpls.2017.01843.10.3389/fpls.2017.01843 PMC5694560 29184557

[75] Soriano JM, Alvaro F (2019). Discovering consensus genomic regions in wheat for root-related traits by QTL meta-analysis. Sci Rep.

[76] Nadolska-Orczyk A, Rajchel IK, Orczyk W, Gasparis S (2017). Major genes determining yield-related traits in wheat and barley. Theor Appl Genet.

[77] Sajjad M, Ma X, Habibullah Khan S, Shoaib M, Song Y, Yang W, Zhang A, Liu D. *TaFlo2-A1*, an ortholog of rice *Flo2*, is associated with thousand grain weight in bread wheat (*Triticum aestivum* L.). BMC Plant Biology. 2017; 17(164): 1–11. 10.1186/s12870-017-1114-3.10.1186/s12870-017-1114-3 PMC5644068 29037166

[78] Zhang L, Zhao YL, Gao LF, Zhao GY, Zhou RH, Zhang BS, Jia JZ. (2012). *TaCKX6-D1*, the ortholog of rice *OsCKX2*, is associated with grain weight in hexaploid wheat. New Phytologist. 2012; 195(3): 574–584. 10.1111/j.1469-8137.2012.04194.x.10.1111/j.1469-8137.2012.04194.x 22670578

[79] Hanif M, Gao F, Liu J, Wen W, Zhang Y, Rasheed A, Xia X, He Z, Cao S (2016). *TaTGW6-A1*, an ortholog of rice *TGW6*, is associated with grain weight and yield in bread wheat. Mol Breeding.

[80] Ma M, Wang Q, Li Z, Cheng H, Li Z, Liu X, Song W, Appels R, Zhao H. Expression of *TaCYP78A3*, a gene encoding cytochrome P450 CYP78A3 protein in wheat (*Triticum aestivum* L.), affects seed size. Plant Journal. 2015; 83(2): 312–325. 10.1111/tpj.12896.10.1111/tpj.12896 26043144

[81] Guo L, Ma M, Wu L, Zhou M, Li M, Wu B, Li L, Liu X, Jing R, Chen W, Zhao H. Modified expression of *TaCYP78A5* enhances grain weight with yield potential by accumulating auxin in wheat (*Triticum aestivum* L.). Plant Biotechnology Journal. 2021; 20(1): 168–182. 10.1111/pbi.13704 10.1111/pbi.13704 PMC8710830 34510688

[82] Zhang K, Wang J, Zhang L, Rong C, Zhao F, Peng T, Li H, Cheng D, Liu X, Qin H, Zhang A, Tong Y, Wang D (2013). Association Analysis of Genomic Loci Important for Grain Weight Control in Elite Common Wheat Varieties Cultivated with Variable Water and Fertiliser Supply. PLoS ONE.

[83] Jones BH, Blake NK, Heo HY, Martin JM, Torrion JA, Talbert LE (2020). Allelic response of yield component traits to resource availability in spring wheat. Theor Appl Genet.

[84] E Z, Zhang Y, Li T, Wang L, Zhao H. Characterization of the ubiquitin-conjugating enzyme gene family in rice and evaluation of expression profiles under abiotic stresses and hormone treatments. PLoS ONE. 2015; 10(4): e0122621. 10.1371/journal.pone.0122621.10.1371/journal.pone.0122621 PMC4406754 25902049

[85] Mathan J, Singh A, Ranjan A (2021). Sucrose transport in response to drought and salt stress involves ABA-mediated induction of *OsSWEET13* and *OsSWEET15* in rice. Physiol Plant.

[86] Gao Y, Wang ZY, Kumar V, Xu XF, Yuan DP, Zhu XF, Li TY, Jia B, Xuan YH (2018). Genome-wide identification of the *SWEET* gene family in wheat. Gene.

[87] Gautam T, Saripalli G, Gahlaut V, Kumar A, Sharma PK, Balyan HS, Gupta PK. Further studies on sugar transporter (*SWEET*) genes in wheat (*Triticum aestivum* L.). Molecular Biology Reports. 2019; 46: 2327–2353. 10.1007/s11033-019-04691-0.10.1007/s11033-019-04691-0 30830588

[88] Mizuta Y, Harushima Y, Kurata N (2010). Rice pollen hybrid incompatibility caused by reciprocal gene loss of duplicated genes. Proc Natl Acad Sci USA.

[89] Abe Y, Mieda K, Ando T, Kono I, Yano M, Kitano H, Iwasaki Y (2010). The SMALL AND ROUND SEED1 (*SRS1/DEP2*) gene is involved in the regulation of seed size in rice. Genes Genet Syst.

[90] Liu Y, Xia X, He Z (2013). Characterization of Dense and Erect Panicle 1 Gene (*TaDep1*) Located on Common Wheat Group 5 Chromosomes and Development of Allele-Specific Markers. Acta Agron Sin.

[91] Xu H, Zhang R, Wang M, Li L, Yan L, Wang Z, Zhu J, Chen X, Zhao A, Su Z, Xing J, Sun Q, Ni Z (2021). Identification and characterization of QTL for spike morphological traits, plant height and heading date derived from the D genome of natural and resynthetic allohexaploid wheat. Theor Appl Genet.

[92] Aoi Y, Hira H, Hayakawa Y, Liu H, Fukui K, Dai X, Tanaka K, Hayashi K, Zhao Y, Kasahara H (2020). UDP-glucosyltransferase UGT84B1 regulates the levels of indole-3-acetic acid and phenylacetic acid in Arabidopsis. Biochem Biophys Res Commun.

[93] Yang D, Li M, Liu Y, Chang L, Cheng H, Chen J, Chai S (2016). Identification of quantitative trait loci and water environmental interactions for developmental behaviors of leaf greenness in wheat. Front Plant Sci.

[94] Li M, Liu Y, Ma J, Zhang P, Wang C, Su J, Yang D. Genetic dissection of stem WSC accumulation and remobilization in wheat (*Triticum aestivum* L.) under terminal drought stress. BMC Genetics. 2020; 21(50): 1–14. 10.1186/s12863-020-00855-1.10.1186/s12863-020-00855-1 PMC7191701 32349674

[95] Yang D, Liu Y, Cheng H, Chang L, Chen J, Chai S, Li M. Genetic dissection of flag leaf morphology in wheat (*Triticum aestivum* L.) under diverse water regimes. BMC Genetics. 2016; 17(94); 1–15. 10.1186/s12863-016-0399-9.10.1186/s12863-016-0399-9 PMC4924301 27352616

[96] Zadoks JC, Chang TT, Konzak CF (1974). A decimal code for the growth stages of cereals. Weed Res.

[97] Toker C. Estimates of broad-sense heritability for seed yield and yield criteria in faba bean (*Vicia faba* L.). Hereditas. 2004; 140(3): 222–225. 10.1111/j.1601-5223.2004.01780.x.10.1111/j.1601-5223.2004.01780.x 15198712

[98] Yang D, Zhang G, Li X, Xin H, Chen H, Ni S, Chen X (2012). Genetic characteristics associated with drought tolerance of plant height and thousand-grain mass of recombinant inbred lines of wheat. Chin J Appl Ecol.

[99] Meng L, Li H, Zhang L, Wang J (2015). QTL IciMapping: Integrated software for genetic linkage map construction and quantitative trait locus mapping in biparental populations. Crop Journal.

[100] Sosnowski O, Charcosset A, Joets J (2012). Biomercator V3: An upgrade of genetic map compilation and quantitative trait loci meta-analysis algorithms. Bioinformatics.

[101] Darvasi A, Soller M (1997). A simple method to calculate resolving power and confidence interval of QTL map location. Behav Genet.

[102] Veyrieras JB, Goffinet B, Charcosset A (2007). MetaQTL: A package of new computational methods for the meta-analysis of QTL mapping experiments. BMC Bioinformatics.

[103] Arcade A, Labourdette A, Falque M, Mangin B, Chardon F, Charcosset A, Joets J (2004). BioMercator: Integrating genetic maps and QTL towards discovery of candidate genes. Bioinformatics.

[104] Borrill P, Ramirez-Gonzalez R, Uauy C (2016). expVIP: A customizable RNA-seq data analysis and visualization platform. Plant Physiol.

[105] Ramírez-González RH, Borrill P, Lang D, Harrington SA, Brinton J, Venturini L, Davey M, Jacobs J, Van Ex F, Pasha A, Khedikar Y, Robinson SJ, Cory AT, Florio T, Concia L, Juery C, Schoonbeek H, Steuernagel B, Xiang D, Uauy C (2018). The transcriptional landscape of polyploid wheat. Science.

