# Supplementary material for: Identification of quantitative trait loci (QTL) and meta-QTL analysis for kernel size-related traits in wheat (Triticum aestivum L.)
Source: BMC Plant Biol. 2022 Dec 23;22:607. doi: 10.1186/s12870-022-03989-9 (PMC9784057; doi:10.1186/s12870-022-03989-9)
Supplement: Supplementary file 1 — Additional file 1: Fig. S1. The rainfall records (mm) for each growing season in seven tested environments. E1-E3 are the experimental environments in Yuzhong farm station during 2015-2016 under DS and WW conditions and during 2016-2017 under DS conditions, respectively. E4-E7 are the experimental environments in Tongwei farm station during 2017-2018 under DS and WW conditions and 2018-2020 under DS conditions, respectively. Fig. S2.Distribution of the markers on the consensus map used for MQTL analysis in thisstudy. [file 12870_2022_3989_MOESM1_ESM.docx]

Fig. S1


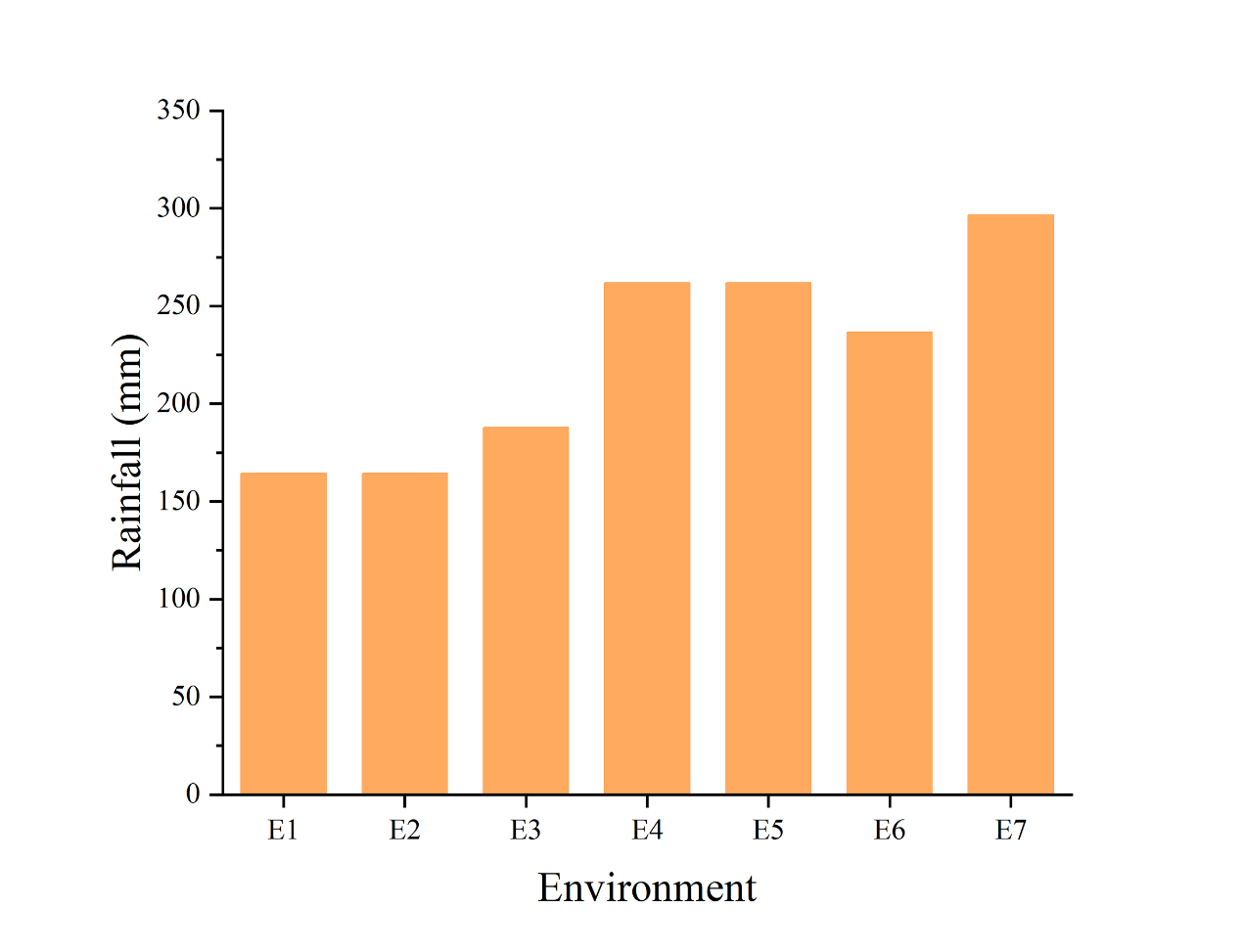


**Fig. S1.** The rainfall records (mm) for each growing season in seven tested environments. E1-E3 are the experimental environments in Yuzhong farm station during 2015-2016 under DS and WW conditions and during 2016-2017 under DS conditions, respectively. E4-E7 are the experimental environments in Tongwei farm station during 2017-2018 under DS and WW conditions and 2018-2020 under DS conditions, respectively.

Fig. S2


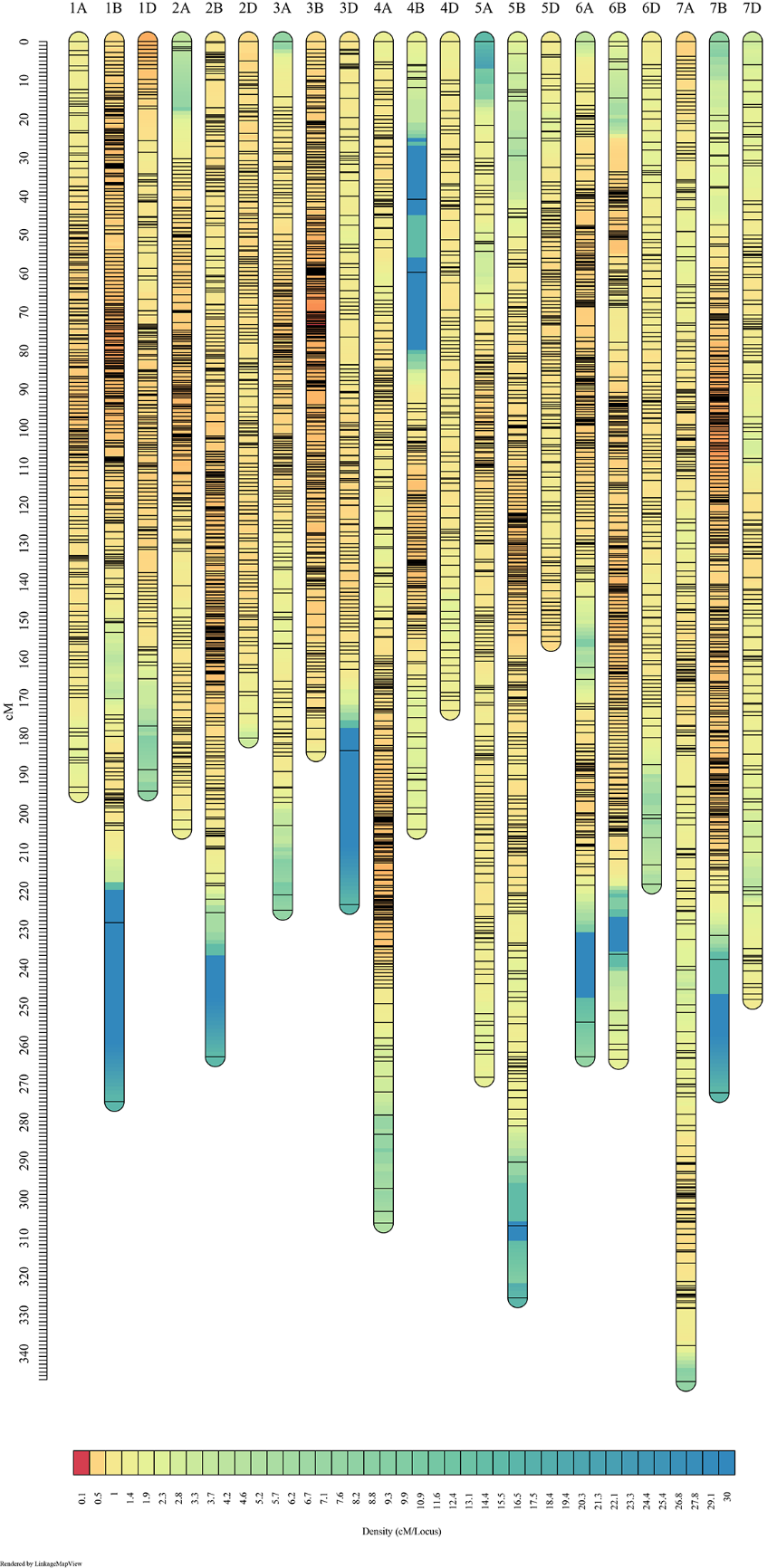


**Fig. S2.** Distribution of the markers on the consensus map used for MQTL analysis in this study.
